# Association of Sodium-Glucose Cotransporter-2 Inhibitors With Incident Atrial Fibrillation in Older Adults With Type 2 Diabetes

**DOI:** 10.1001/jamanetworkopen.2022.35995

**Published:** 2022-10-11

**Authors:** Min Zhuo, Elvira D’Andrea, Julie M. Paik, Deborah J. Wexler, Brendan M. Everett, Robert J. Glynn, Seoyoung C. Kim, Elisabetta Patorno

**Affiliations:** 1 Division of Pharmacoepidemiology and Pharmacoeconomics, Brigham and Women’s Hospital, Boston, Massachusetts; 2 Division of Renal Medicine, Brigham and Women’s Hospital, Boston, Massachusetts; 3 Department of Medicine, Brigham and Women’s Hospital and Harvard Medical School, Boston, Massachusetts; 4 Division of Nephrology, Department of Medicine, Beth Israel Deaconess Medical Center and Harvard Medical School, Boston, Massachusetts; 5 New England Geriatric Research, Education and Clinical Center, VA Boston Healthcare System, Boston, Massachusetts; 6 Diabetes Center, Massachusetts General Hospital and Harvard Medical School, Boston; 7 Divisions of Cardiovascular and Preventive Medicine, Brigham and Women’s Hospital, Boston, Massachusetts; 8 Division of Rheumatology, Inflammation, and Immunity, Brigham and Women’s Hospital, Boston, Massachusetts

## Abstract

**Importance:**

Sodium-glucose cotransporter-2 inhibitors (SGLT-2is) have demonstrated many cardiovascular and kidney function benefits for patients with type 2 diabetes (T2D). However, the results of SGLT-2i use in primary prevention of atrial fibrillation (AF) were inconsistent in clinical trials, and incident AF was not a prespecified end point.

**Objective:**

To examine incident AF with initiation of an SGLT-2i compared with initiation of a dipeptidyl peptidase-4 inhibitor (DPP-4i) or a glucagonlike peptide-1 receptor agonist (GLP-1RA) among older adults (aged ≥66 years) with T2D in routine clinical practice.

**Design, Setting, and Participants:**

A population-based new-user cohort study included older adults with T2D who had no history of AF and were enrolled in Medicare fee-for-service from April 1, 2013, to December 31, 2018. Data analysis was performed from June 28 to December 1, 2021.

**Exposures:**

To control for potential confounding, new users of SGLT-2i were 1:1 propensity score (PS)–matched to new users of DPP-4is or GLP-1RAs in 2 pairwise comparisons based on 138 baseline covariates.

**Main Outcomes and Measures:**

The primary outcome was incident AF, defined as an inpatient diagnosis code for AF. Hazard ratios (HRs) and rate differences (RDs) per 1000 person-years, with their 95% CIs, were estimated in the PS-matched groups.

**Results:**

New users of SGLT-2is were 1:1 PS-matched to new users of a DPP-4i (n = 74 868) or GLP-1RA (n = 80 475). Overall, the mean (SD) age of study participants was 72 (5) years, and 165 984 were women (53.4%). The risk of incident AF was lower in the SGLT-2i group than the matched DPP-4i group (HR, 0.82; 95% CI, 0.76 to 0.89; RD, –3.7; 95% CI, –5.2 to –2.2 per 1000 person-years) or the matched GLP-1RA group (HR, 0.90; 95% CI, 0.83 to 0.98; RD, –1.8; 95% CI, –3.2 to –0.3 per 1000 person-years). Results were consistent across several sensitivity and subgroup analyses.

**Conclusions and Relevance:**

The findings of this study suggest that the initiation of an SGLT-2i was associated with a reduced risk of incident AF compared with a DPP-4i or GLP-1RA. The results may be helpful when weighing the potential risks and benefits of various glucose level–lowering agents in older adults with T2D.

## Key Points

**Question:**

Is use of sodium-glucose cotransporter-2 inhibitors (SGLT-2is) associated with reduced risk of atrial fibrillation (AF) in older patients with type 2 diabetes?

**Findings:**

In this US nationwide cohort study assessing the risk of AF among more than 200 000 Medicare beneficiaries with type 2 diabetes, after propensity score matching, the initiation of SGLT-2is was associated with an 18% decrease in the risk of AF compared with dipeptidyl peptidase-4 inhibitors and a 10% decrease in the risk of AF compared with glucagon-like peptide-1 receptor agonists.

**Meaning:**

These results provide substantive evidence supporting the potential benefits of initiation of an SGLT-2i in older adults with type 2 diabetes.

## Teaser

This cohort study compares the incidence of atrial fibrillation in patients with type 2 diabetes associated with use of sodium-glucose cotransporter-2 inhibitors with dipeptidyl peptidase-4 inhibitors or glucagonlike peptide-1 receptor agonists.

## Introduction

More than 30 million individuals (10%) in the US have type 2 diabetes (T2D), with a higher prevalence—more than 1 in 4—among adults aged 65 years or older.^1^ Type 2 diabetes is associated with a 35% to 60% relative increase in the risk of developing atrial fibrillation (AF) or atrial flutter (collectively referred to as AF).^2,3,4^ Compared with T2D alone, the presence of comorbid T2D and new-onset AF carries a 3.8-fold increased risk for heart failure (HF) and a 2.7-fold increased risk for all-cause mortality.^5^ Among patients older than 65 years, 27% develop AF in 10 years.^6^ Owing to the high risk of developing AF, preventing this condition in older patients with T2D is an important goal.

Large randomized clinical trials have proven the efficacy of sodium-glucose cotransporter-2 inhibitors (SGLT-2is) in reducing major cardiovascular events, hospitalization for HF, and kidney disease progression.^7,8,9^ However, the role of SGLT-2is in incident AF remains controversial. In the DECLARE-TIMI 58 trial, compared with placebo, dapagliflozin reduced the incidence of AF by 19% in patients with T2D.^10^ However, there was no consistent reduction in incident AF with SGLT-2is in other randomized clinical trials.^7,9,11,12,13^ Meta-analyses suggested either no effect or possible protective effects of SGLT-2is against AF.^13,14,15,16,17,18^However, incident AF was not a prespecified end point and was typically reported as an adverse event in randomized clinical trials. This reporting generally depends on investigators identifying events and reporting them in a relatively unstructured way.^19^ In addition, incident AF was not a common event in randomized clinical trials, further limiting the ability to assess this outcome robustly. Head-to-head trials comparing SGLT-2is with other antidiabetes drugs also were lacking. Thus, we sought to quantify SGLT-2i initiation with incident AF compared with 2 active comparators in a nationwide cohort of older adults with T2D.

## Methods

### Study Design and Data Sources

We conducted a population-based cohort study using Medicare fee-for-service data. We used Medicare claims data from inpatient services (Part A), outpatient services (Part B), and prescription medications (Part D).

The initiators of SGLT-2is were compared with the initiators of dipeptidyl peptidase-4 inhibitors (DPP-4is) or glucagonlike peptide-1 receptor agonists (GLP-1RAs) in 2 pairwise comparisons. Any of these 3 antidiabetes drug classes may have been selected as a second-line treatment for T2D per clinical guidelines available during the study period.^20,21^ This study was approved by the Mass General Brigham Institutional Review Board, and an appropriate data use agreement was in place. Informed consent was not obtained because the study used claims data with anonymous identifiers. Data analysis was performed from June 28 to December 1, 2021. This study followed the Strengthening the Reporting of Observational Studies in Epidemiology (STROBE) reporting guideline for observational studies.

### Study Population

We identified patients who newly filled a prescription for an SGLT-2i or a comparator between April 1, 2013 (date after the first SGLT-2i approval in the US), and December 31, 2018 (end date for available data). For each pairwise comparison, the cohort entry date was the date of the first prescription of an SGLT-2i or the specific comparator during the study period. Eligible patients had at least 365 days of continuous Medicare Part A, B, and D enrollment before cohort entry. We excluded patients with prior use of an SGLT-2i or the specific comparator in the 365-day baseline period. We restricted the study population to patients with T2D aged 66 years or older. Patients with missing demographic information (age, sex, or race) were excluded because these data may influence outcome. Race and ethnicity data in Medicare are derived from source data from the Social Security Administration and the results of an algorithm that applies to the source data; race data were included in the analyses as demographic characteristics. We also excluded those who had a diagnosis of any of the following during the baseline period: type 1 diabetes, secondary diabetes, cancer, chronic kidney disease stage 5 or dialysis, organ transplant, nursing home admission, prior AF, or factors suggestive of AF.^22^ Codes for inclusion and exclusion criteria are reported in eTable 1 in the Supplement.

### Follow-up and Outcomes

Follow-up began on the day after cohort entry and continued in an as-treated scheme until the first occurrence of any of the following: an outcome event, death, switching to a comparator class, discontinuation of index therapy, end of the study period, or end of health care or pharmacy enrollment. Medication use was evaluated by prescription refill date and supply. Treatment discontinuation was defined as not refilling a prescription within 60 days after the most recent filled prescription days supply ran out.

The primary outcome was incident AF, defined as an inpatient diagnosis code for AF (henceforth termed AF hospitalization) using a previously validated algorithm that has 95% sensitivity and 99% specificity.^23^ Secondary outcomes included AF diagnosis (based on at least 1 inpatient or 2 outpatient diagnosis codes for AF),^24,25^ any AF diagnosis code combined with dispensing of any AF medication within 30 days (henceforth termed *AF treated with medication*),^26^ and hospitalization for AF (defined as AF discharge diagnosis codes in the primary position). Other secondary outcomes included stroke or transient ischemic attack, hospitalization for HF (HF discharge diagnosis codes in the primary position), AF hospitalization censored for HF, and hospitalization for AF and HF (AF and HF discharge diagnosis codes in any position). Codes for outcomes are available in eTable 2 in the Supplement.

### Statistical Analyses

To account for the nonrandom allocation of patients to the treatment groups, we used 1:1 propensity score (PS) matching. Propensity scores were calculated using multivariable logistic regression that modeled the probability of initiating an SGLT-2i vs a comparator as a function of 138 predefined baseline covariates. Covariate selection was based on the extent of our knowledge that these covariates were either confounders or risk factors for the outcome. These covariates were assessed during a 365-day period before the cohort entry date and included demographic characteristics (eg, age, sex, or race), year of cohort entry, comorbid conditions (eg, chronic kidney disease, hypertension, or HF), drug therapy (eg, anticoagulants or β-blockers), and health care use (eg, electrocardiogram, hospitalization, or cardiologist visit). To further quantify the burden of comorbidities, we calculated a claims-based frailty index^27^ and a combined comorbidity score.^28^ For each of the 2 pairwise comparisons, we created a 1:1 PS-matched cohort using a nearest-neighbor matching without replacement approach within a maximum caliper width of 0.01.^29^ We assessed covariate balance among the matched cohorts by using standardized mean differences, with values less than 0.1 suggesting an adequate balance between matched groups.^30^

For all outcomes in each PS-matched cohort, we calculated the incidence rates (IRs) as well as hazard ratios (HRs) using Cox proportional hazards regression models and rate differences (RDs) using a weighted least-squares regression approach,^31^ each with 95% CIs. For the primary outcome, we produced Kaplan-Meier plots of cumulative incidence and compared IRs between treatment groups with log-rank tests.

We performed several prespecified sensitivity analyses. We changed the grace period and risk period from 60 days to 30 days and from 60 days to 90 days. In addition to the primary as-treated analysis, we carried the index exposure forward to 365 days without considering treatment discontinuation or switching to mimic an intention-to-treat approach. To assess the presence of potential unmeasured confounding, we evaluated the association of SGLT-2i use with the risk for herpes zoster previously reported to be unrelated to this drug class.^32^

We also quantified the association of SGLT-2is and AF in several relevant subgroups: (1) age 70 years or younger vs older than 70 years, (2) female vs male, (3) no history of HF vs a history of HF, and (4) no history of atherosclerotic cardiovascular disease vs a history of atherosclerotic cardiovascular disease.

*P* values <.05 (paired, 2-sided) were considered statistically significant. All analyses were performed using Aetion Evidence Platform (2020), version R4.34 software^33^ for data analysis.^34,35^

## Results

### Study Cohort and Patient Characteristics

A total of 408 294 patients met study criteria for the SGLT-2i vs DPP-4i cohort (82 430 SGLT-2i users and 325 864 DPP-4i users) and 234 530 met the criteria for the SGLT-2i vs GLP-1RA cohort (121 371 SGLT-2i users and 113 159 GLP-1RA users) (eFigure 1 and eFigure 2 in the Supplement). Before PS-matching, patients initiating an SGLT-2i had lower frailty and combined comorbidity scores^28^(eTable 3 and eTable 4 in the Supplement).

After 1:1 PS-matching (*c* statistic of 0.5 for both models), we identified 149 736 patients (74 868 pairs) initiating either an SGLT-2i or DPP-4i and 160 950 patients (80 475 pairs) initiating either an SGLT-2i or GLP-1RA. The mean (SD) age was 72 (5) years, 165 984 were women (53.4%), and 144 702 were men (46.6%) (selected baseline characteristics in Table 1). In the matched SGLT-2i vs DPP-4i cohort, 41 830 of SGLT-2i patients (55.9%) initiated canagliflozin, followed by empagliflozin (19 779 [26.5%]) and dapagliflozin (13 259 [17.7%]). Similarly, in the matched SGLT-2i vs GLP-1RA cohort, 44 085 of SGLT-2i patients (54.8%) initiated canagliflozin, followed by empagliflozin (21 699 [27.0%]) and dapagliflozin (14 691 [18.3%]). After PS-matching, the median duration of follow-up was 191 (IQR, 90-401) days among SGLT-2i users and 214 (IQR, 116-438) days among DPP-4i users in the SGLT-2i vs DPP-4i cohort and 188 (IQR, 90-395) days among SGLT-2i users and 173 (IQR, 88-374) days among GLP-1RA users in the SGLT-2i vs GLP-1RA cohort.

**Table 1.** Selected Baseline Characteristics of SGLT-2i vs DPP-4i and SGLT-2i vs GLP-1RA Cohorts After 1:1 Propensity Score Matching

Characteristic	SGLT-2i vs DPP-4i (n = 74 868 pairs)			SGLT-2i vs GLP-1RA (n = 80 475 pairs)		
	Patients, No. (%)		Standardized difference	Patients, No. (%)		Standardized difference
	SGLT-2i	DPP-4i		SGLT-2i	GLP-1RA	
Age, mean (SD), y	71.8 (5.0)	71.7 (5.1)	0.02	71.8 (5.1)	71.8 (5.1)	0
Sex						
Female	38 566 (51.5)	38 470 (51.4)	0	44 498 (55.3)	44 450 (55.2)	0
Male	36 302 (48.5)	36 398 (48.6)	0	35 977 (44.7)	36 025 (44.8)	0
Race						
Black	5904 (7.9)	5985 (8.0)	0	6581 (8.2)	6616 (8.2)	0
White	61 904 (82.7)	61 794 (82.5)	0.01	66 883 (83.1)	66 583 (82.7)	0.01
Other^a^	7060 (9.4)	7089 (9.5)	0	7011 (8.7)	7276 (9.0)	−0.01
Diabetes-related comorbidities						
Diabetic						
Nephropathy	8293 (11.1)	8333 (11.1)	0	10 960 (13.6)	11 044 (13.7)	0
Neuropathy	18 495 (24.7)	18 420 (24.6)	0	21 951 (27.3)	21 972 (27.3)	0
Retinopathy	9930 (13.3)	9816 (13.1)	0.01	11 586 (14.4)	11 656 (14.5)	0
Hyperglycemia	25 879 (34.6)	26 023 (34.8)	−0.01	30 568 (38.0)	30 289 (37.6)	0.01
Hypoglycemia	6251 (8.3)	6126 (8.2)	0	7230 (9.0)	7212 (9.0)	0
Other comorbid condition						
Hyperthyroidism	778 (1.0)	803 (1.1)	−0.01	905 (1.1)	916 (1.1)	0
Chronic kidney disease stages 3-4	5896 (7.9)	5763 (7.7)	0.01	8636 (10.7)	8780 (10.9)	−0.01
Hypertension	68 604 (91.6)	68 639 (91.7)	0	74 383 (92.4)	74 397 (92.4)	0
Coronary atherosclerosis	21 324 (28.5)	21 302 (28.5)	0	22 927 (28.5)	22 827 (28.4)	0
Cardiac conduction disorder^b^	3157 (4.2)	3173 (4.2)	0	3494 (4.3)	3537 (4.4)	0
Other cardiac dysrhythmia^c^	7227 (9.7)	7229 (9.7)	0	7904 (9.8)	7887 (9.8)	0
Cardiomyopathy	1764 (2.4)	1732 (2.3)	0.01	1943 (2.4)	1922 (2.4)	0
Congestive heart failure	5907 (7.9)	5865 (7.8)	0	6883 (8.6)	6864 (8.5)	0
Valve disorders	7508 (10.0)	7449 (9.9)	0	8240 (10.2)	8240 (10.2)	0
Other cardiovascular disease^d^	8292 (11.1)	8237 (11.0)	0	9262 (11.5)	9295 (11.6)	0
Transient ischemic attack	1710 (2.3)	1689 (2.3)	0	1928 (2.4)	1917 (2.4)	0
Ischemic stroke	7730 (10.3)	7758 (10.4)	0	8509 (10.6)	8496 (10.6)	0
Peripheral artery disease	8505 (11.4)	8531 (11.4)	0	9841 (12.2)	9853 (12.2)	0
Alcohol abuse or dependence	860 (1.1)	854 (1.1)	0	832 (1.0)	815 (1.0)	0
Overweight	6133 (8.2)	6311 (8.4)	0	6522 (8.1)	6483 (8.1)	0
Obesity	24 452 (32.7)	24 383 (32.6)	0	29 408 (36.5)	29 319 (36.4)	0
Claims-based frailty index						
Nonfrail	25 196 (33.7)	25 506 (34.1)	−0.01	23 802 (29.6)	23 779 (29.5)	0
Prefrail	43 438 (58.0)	43 094 (57.6)	0.01	48 716 (60.5)	48 669 (60.5)	0
Frail	6234 (8.3)	6268 (8.4)	0	7957 (9.9)	8027 (10.0)	0
Combined comorbidity index, mean (SD)	1.0 (1.8)	1.0 (1.8)	0	1.2 (1.9)	1.2 (1.9)	0
Use of diabetes drugs						
Metformin	59 258 (79.1)	59 503 (79.5)	−0.01	61 810 (76.8)	61 816 (76.8)	0
Sulfonylureas	34 628 (46.3)	34 814 (46.5)	0	39 270 (48.8)	39 280 (48.8)	0
Insulin	20 174 (26.9)	20 112 (26.9)	0	25 194 (31.3)	25 180 (31.3)	0
GLP-1RA	8686 (11.6)	8114 (10.8)	0.03			
DPP-4i				29 216 (36.3)	29 545 (36.7)	−0.01
No. of antidiabetic medications at index date, mean (SD)	2.3 (0.9)	2.3 (0.8)	0	2.5 (1.0)	2.5 (1.0)	0
Other drugs						
Antiarrhythmics	91 (0.1)	96 (0.1)	0	97 (0.1)	106 (0.1)	0
Anticoagulants	796 (1.1)	802 (1.1)	0	879 (1.1)	875 (1.1)	0
Angiotensin-converting enzyme inhibitors	34 519 (46.1)	34 580 (46.2)	0	37 156 (46.2)	37 080 (46.1)	0
Angiotensin II receptor blockers	26 959 (36.0)	26 801 (35.8)	0	30 279 (37.6)	30 455 (37.8)	0
β-blockers	31 674 (42.3)	31 645 (42.3)	0	35 054 (43.6)	34 988 (43.5)	0
Calcium channel blockers	24 164 (32.3)	23 959 (32.0)	0.01	26 754 (33.2)	26 710 (33.2)	0
Antiplatelet agents	9711 (13.0)	9777 (13.1)	0	10 632 (13.2)	10 646 (13.2)	0
Statins	57 809 (77.2)	57 683 (77.0)	0	63 174 (78.5)	63 169 (78.5)	0
No. of total medications, mean (SD)	12.6 (5.7)	12.6 (5.8)	0	13.6 (6.1)	13.6 (5.8)	0
Health care use						
Electrocardiogram	33 390 (44.6)	33 344 (44.5)	0	36 342 (45.2)	36 337 (45.2)	0
Cardiac imaging	17 791 (23.8)	17 702 (23.6)	0	19 578 (24.3)	19 620 (24.4)	0
HbA_1c_ test order	72 440 (96.8)	72 426 (96.7)	0.01	78 054 (97.0)	78 042 (97.0)	0
Metabolic panel test	71 881 (96.0)	71 923 (96.1)	−0.01	77 204 (95.9)	77 246 (96.0)	−0.01
Emergency department visit	18 739 (25.0)	18 740 (25.0)	0	21 157 (26.3)	21 214 (26.4)	0
Hospitalization	7113 (9.5)	7148 (9.5)	0	7880 (9.8)	7888 (9.8)	0
Internal medicine visit	66 533 (88.9)	66 620 (89.0)	0	71 508 (88.9)	71 537 (88.9)	0
Cardiologist visit	29 731 (39.7)	29 597 (39.5)	0	32 550 (40.4)	32 512 (40.4)	0
Endocrinologist visit	13 832 (18.5)	13 600 (18.2)	0.01	17 389 (21.6)	17 450 (21.7)	0.
Nephrologist visit	2927 (3.9)	2958 (4.0)	−0.01	4199 (5.2)	4376 (5.4)	−0.01
No. of office visits, mean (SD)	10.3 (7.1)	10.3 (7.3)	0	11.0 (7.5)	11.0 (7.4)	0

Abbreviations: DPP-4i, dipeptidyl peptidase-4 inhibitor; HbA_1c_, hemoglobin A_1c_; GLP-1RA, glucagonlike peptide-1 receptor agonist; SGLT-2i, sodium-glucose cotransporter-2 inhibitors.

^a^ Other includes Asian, North American Native, Hispanic, and other (plus an unknown category).

^b^ Cardiac conduction disorder includes atrioventricular block, left bundle branch block, and right bundle branch block.

^c^ Other cardiac arrhythmia includes ventricular tachycardia and premature beats.

^d^ Other cardiovascular disease includes rheumatic heart disease, pericarditis, and myocarditis.

### Primary Outcome

**Figure 1.**
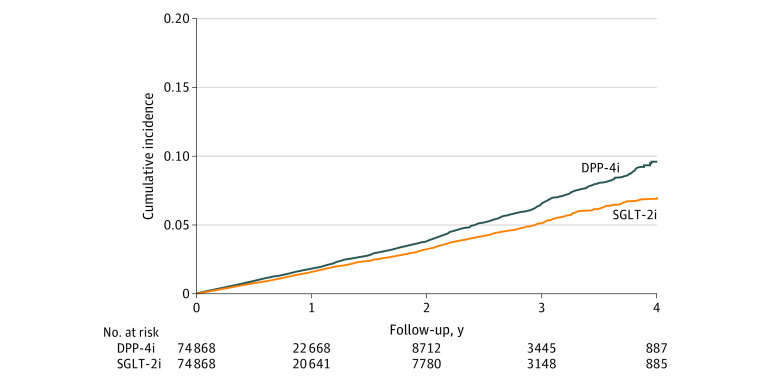
Cumulative Incidence of Atrial Fibrillation in Propensity Score–Matched Sodium-Glucose Cotransporter-2 Inhibitor (SGLT-2i) vs Dipeptidyl Peptidase-4 Inhibitor (DPP-4i) Cohort Hazard ratio, 0.82 (95% CI, 0.76-0.89).

**Figure 2.**
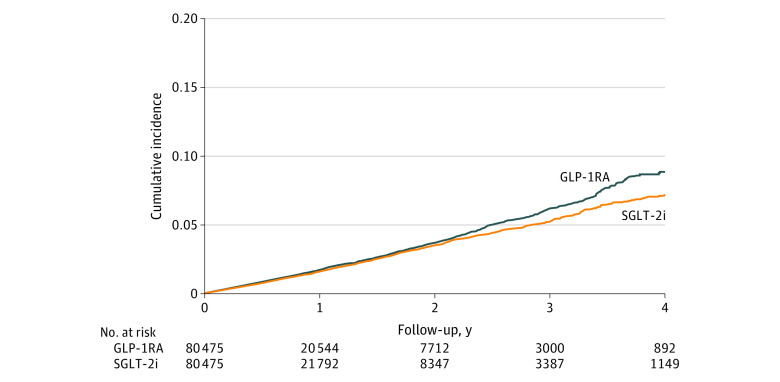
Cumulative Incidence of Atrial Fibrillation in Propensity Score–Matched Sodium-Glucose Cotransporter-2 Inhibitor (SGLT-2i) vs Glucagonlike Peptide-1 Receptor Agonist (GLP-1RA) Cohort Hazard ratio, 0.90 (95% CI, 0.83-0.98).

Reasons for censoring in the matched cohorts are reported in eTable 5 in the Supplement. After PS-matching, there were 1082 AF hospitalization events among SGLT-2i users and 1410 events among DPP-4i users in the SGLT-2i vs DPP-4i cohort (IR, 16.8 vs 20.5 per 1000 person-years; HR, 0.82; 95% CI, 0.76 to 0.89; and RD, –3.7; 95% CI, –5.2 to –2.2 per 1000 person-years) and 1175 events among SGLT-2i users and 1235 events among GLP-1RA users in the SGLT-2i vs GLP-1RA cohort (IR, 17.0 vs 18.7 per 1000 person-years; HR, 0.90; 95% CI, 0.83 to 0.98; RD, –1.8; 95% CI, –3.2 to –0.3 per 1000 person-years), as reported in Table 2. Kaplan-Meier plots comparing the cumulative incidence of AF over time in the matched groups are shown in Figure 1 and Figure 2.

**Table 2.** Primary and Secondary Outcome Findings in 1:1 Propensity Score–Matched Initiators of SGLT-2i vs DPP-4i or GLP-1RA

Outcome	No. of events (IR)		SGLT-2i vs DPP-4i (n = 74 868 pairs)		No. of events (IR)		SGLT-2i vs GLP-1RA (n = 80 475 pairs)	
	SGLT-2i	DPP-4i	HR (95% CI)	RD (95% CI)	SGLT-2i	GLP-1RA	HR (95% CI)	RD (95% CI)
Primary outcome								
AF hospitalization	1082 (16.8)	1410 (20.5)	0.82 (0.76 to 0.89)	– 3.7 (– 5.2 to – 2.2)	1175 (17.0)	1235 (18.7)	0.90 (0.83 to 0.98)	– 1.8 (– 3.2 to – 0.3)
Secondary outcomes								
AF diagnosis	1354 (21.1)	1707 (24.9)	0.85 (0.79 to 0.91)	– 3.8 (– 5.5 to – 2.2)	1438 (20.8)	1562 (23.8)	0.87 (0.81 to 0.94)	– 3.0 (– 4.6 to – 1.4)
AF treated with medication	1098 (17.1)	1339 (19.5)	0.88 (0.81 to 0.95)	– 2.4 (– 3.9 to – 1.0)	1143 (16.5)	1281 (19.5)	0.85 (0.78 to 0.92)	– 3.0 (– 4.4 to – 1.5)
Hospitalization for AF	212 (3.3)	251 (3.6)	0.91 (0.75 to 1.09)	– 0.4 (– 1.0 to 0.3)	211 (3.0)	275 (4.1)	0.73 (0.61 to 0.87)	– 1.3 (– 1.9 to – 0.8)
Stroke or TIA	536 (8.3)	662 (9.5)	0.86 (0.77 to 0.96)	– 1.3 (– 2.3 to – 0.3)	587 (8.4)	552 (8.3)	1.01 (0.90 to 1.14)	0.1 (0.9 to 1.1)
Hospitalization for HF	312 (4.8)	684 (9.9)	0.49 (0.43 to 0.56)	– 5.1 (– 6.0 to – 4.1)	388 (5.6)	519 (7.8)	0.71 (0.62 to 0.81)	– 2.3 (– 3.1 to – 1.4)
AF hospitalization censored for HF hospitalization	988 (13.2)	1215 (16.2)	0.86 (0.79 to 0.93)	– 2.6 (– 4.0 to – 1.2)	1089 (13.5)	1122 (13.9)	0.92 (0.84 to 1.00)	– 3.1 (– 4.2 to – 2.0)
Hospitalization for AF and HF	376 (5.8)	574 (8.3)	0.70 (0.62 to 0.80)	– 2.5 (– 3.4 to – 1.6)	464 (6.7)	525 (7.9)	0.92 (0.83 to 1.01)	– 1.3 (– 2.2 to – 0.4)

Abbreviations: AF, atrial fibrillation; DPP-4i, dipeptidyl peptidase-4 inhibitor; GLP-1RA, glucagonlike peptide-1 receptor agonist; HF, heart failure; HR, hazard ratio; IR, incidence rate in 1000 patient-years; RD, rate difference in 1000 patient-years; SGLT-2i, sodium-glucose cotransporter-2 inhibitor; TIA, transient ischemic attack.

### Sensitivity and Subgroup Analyses

**Figure 3.**
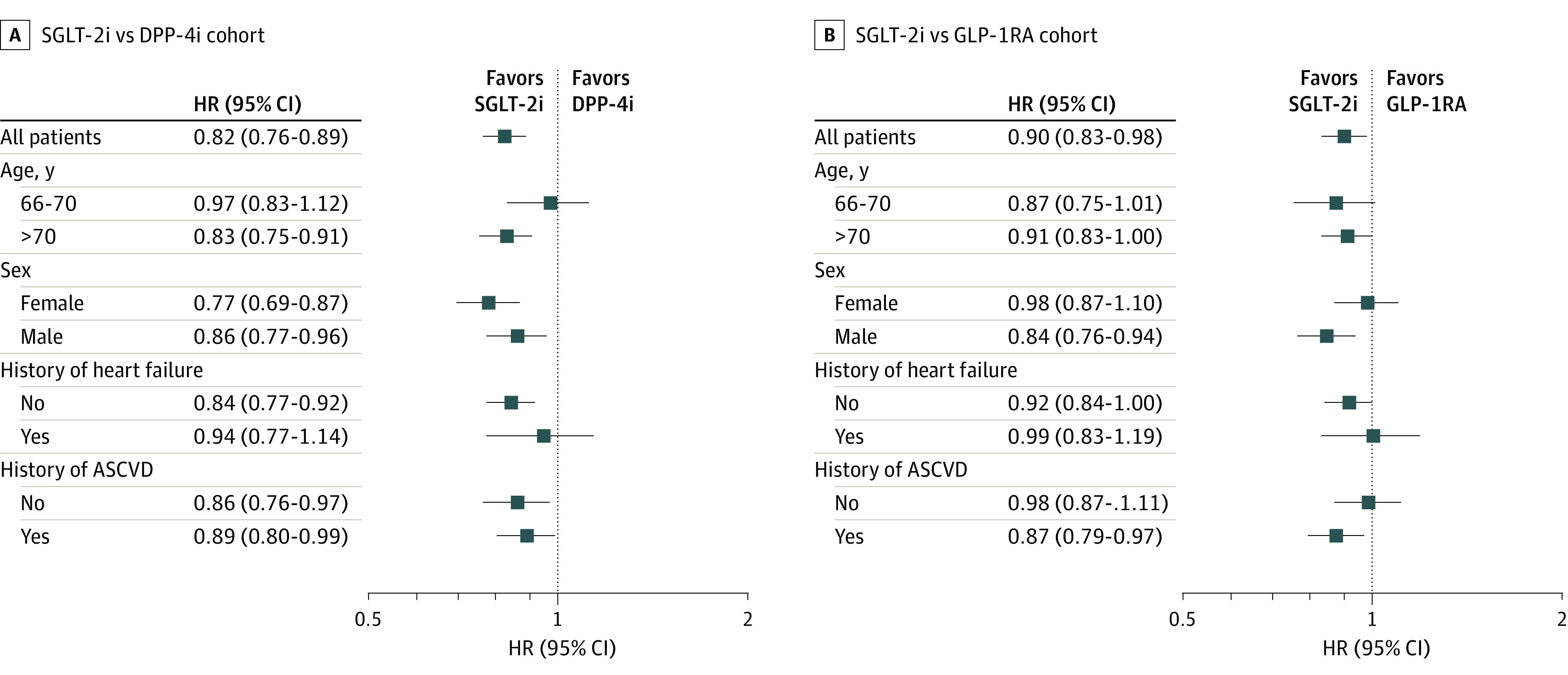
Subgroup Analyses for Incident Atrial Fibrillation in Propensity Score–Matched Cohorts Sodium-glucose cotransporter-2 inhibitor (SGLT-2i) vs dipeptidyl peptidase-4 inhibitor (DPP-4i) (A) and SGLT-2i vs glucagonlike peptide 1 receptor agonist (GLP-1RA cohort (B). ASCVD indicates atherosclerotic cardiovascular disease; HR, hazard ratio.

We performed several sensitivity analyses to assess the robustness of our primary study findings, which produced consistent results. The expected null association with SGLT-2is and the risk for herpes zoster was correctly estimated (eTable 6 in the Supplement). There was no evidence of effect heterogeneity in the association between SGLT-2i and incident AF by age, sex, history of HF, or history of atherosclerotic cardiovascular disease (Figure 3).

### Secondary Outcomes

Risks for AF diagnosis (HR, 0.85; 95% CI, 0.79-0.91), AF treated with medication (HR, 0.88; 95% CI, 0.81-0.95), and hospitalization for AF (HR, 0.91; 95% CI, 0.75-1.09) were also decreased for SGLT-2i users compared with matched DDP-4i users. Risks for AF diagnosis (HR, 0.87; 95% CI, 0.81-0.94), AF treated with medication (HR, 0.85; 95% CI, 0.78-0.92), and hospitalization for AF (HR, 0.73; 95% CI, 0.61-0.87) were also decreased for SGLT-2i users compared with matched GLP-1RA users (Table 2). An association was noted between SGLT-2i use and a lower risk of stroke/transient ischemic attack (HR, 0.86; 95% CI, 0.77-0.96), hospitalization for HF (HR, 0.49; 95% CI, 0.43-0.56), AF hospitalization censored for HF (HR, 0.86; 95% CI, 0.79-0.93), and AF with HF (HR, 0.70; 95% CI, 0.62-0.80) compared with DPP-4is. Compared with GLP-1RAs, SGLT-2is were associated with a similar risk of stroke/transient ischemic attack (HR, 1.01; 95% CI, 0.90-1.14), and a reduced risk of hospitalization for HF (HR, 0.71; 95% CI, 0.62-0.81), AF hospitalization censored for HF (HR, 0.92; 95% CI, 0.84-1.00), and AF with HF (HR, 0.92; 95% CI, 0.83-1.01).

## Discussion

In this nationwide cohort study including more than 200 000 routine-care older patients with T2D, after PS matching, we found that the initiation of SGLT-2is was associated with an 18% reduction in the risk of incident AF compared with DPP-4is and a 10% reduction compared with GLP-1RAs. Study findings were robust to a range of predefined sensitivity analyses and did not appear to differ substantially across subgroups. The SGLT-2is were associated with a lower risk of stroke/transient ischemic attack compared with DPP-4is. Patients initiating SGLT-2i therapy also experienced a decreased risk of hospitalization for HF, AF hospitalization censored for HF, and AF with HF compared with DPP-4i or GLP-1RA initiation.

SGLT-2is have led to a paradigm shift in the management of T2D. However, the effects of SGLT-2is on incident AF in patients with T2D remained unclear from large randomized clinical trials,^7,8,11^ and meta-analyses suggested either no effect or possible protective effects of SGLT-2is against AF.^13,14,15,16,17,18^ Patients with baseline AF were not excluded from randomized clinical trials, and AF events were documented as serious adverse events rather than being a prespecified end point. In addition, patients older than 65 years with multiple comorbidities, who are at the greatest risk for AF, were not meaningfully represented in these randomized clinical trials. Similarly, the association between SGLT-2i use and the risk for AF in routine practice has been primarily evaluated among patients younger than 65 years. In an observational study including patients with T2D in Nordic countries, among 1:3 PS-matched new users (mean age, 61 years) of dapagliflozin (n = 10 227) or a DPP-4i, dapagliflozin was associated with a similar risk of AF as a DPP-4i (HR, 0.92; 95% CI, 0.7-1.12).^36^ A cohort study from Taiwan found that patients with T2D who were prescribed an SGLT-2i (72% aged <60 years) had a similar risk of AF compared with 1:1 PS-matched patients not receiving SGLT-2i therapy.^37^ Another cohort study showed that SGLT-2i use (n = 15 606) was associated with a decreased risk of incident AF (HR, 0.61; 95% CI, 0.50-0.73) compared with PS-weighted DPP-4i users (mean age, 60 years).^38^ Large population-based studies that focus on older adults with multiple comorbidities are lacking. To fill these knowledge gaps, we used a large nationwide sample drawn from 100% US Medicare data, which include health care information on the vast majority of legal US residents aged 65 years or older leading to high precision of the estimates and generalizability. The absolute rate reduction in incident AF we observed among SGLT-2i users in the SGLT-2i vs DPP-4i cohort corresponds to a number needed to treat^39^ for preventing an additional AF event of 435 at 6 months and 250 at 12 months after treatment initiation. In the SGLT-2i vs GLP-1RA group, the number needed to treat^39^ for preventing an additional AF event at 6 months after treatment initiation was 588 and, at 12 months, 263.

Our study has important clinical implications. The latest clinical guidelines for the treatment of T2D recommend SGLT-2is, DPP-4is, and GLP-1RAs as second-line therapies and suggest the choice among these medications should be based on patient-specific characteristics (eg, history of cardiovascular disease).^40^ Type 2 diabetes affects 27% of US adults aged 65 years or older^1^ and is a risk factor for AF,^3^ as is older age, with each decade of advancing age increasing the risk of AF by more than 2-fold.^3^ All treatments for AF are associated with risk, and a number are also associated with high cost.^41^ Therefore, a glucose-lowering medication preventing AF would be advantageous for older adults with T2D. To our knowledge, our study is the first clinical practice investigation to describe the risk of AF in older patients (mean age, 72 years) with T2D who began SGLT-2i therapy. Our results are consistent with a previous study using the US Food and Drug Administration adverse event reporting system, which supports a protective role of SGLT-2is against the occurrence of AF.^42^

To date, the mechanisms through which SGLT-2is could reduce the risk of AF are still under investigation. In addition to potential AF protection through the reduction in risk of HF and atrial stretch, experimental and clinical data have suggested several explanations. It has been postulated that SGLT-2is reduce electrical and structural remodeling of the atrium,^43,44,45^ as well as attenuate the late sodium current-induced calcium overload and arrhythmogenesis.^46^ Furthermore, SGLT-2is improve mitochondrial function,^47^ reverse diabetes-induced sodium/hydrogen exchanger hyperactivity and oxidative stress, stimulate adenosine monophosphate–activated protein kinase activation, and enhance myocardial energetics.^46,48,49^ In addition, SGLT-2is ameliorate many risk factors associated with AF development, such as obesity, hypertension, and hyperglycemia.^16^ Further research is needed to better elucidate the mechanisms of protection against incident AF associated with SGLT-2is.

### Limitations

This study has limitations. First, residual confounding by unmeasured factors cannot be ruled out. For example, socioeconomic status might affect the choice of diabetes treatments owing to the Medicare reimbursement system,^50,51^ and lower socioeconomic status is associated with a higher risk of AF.^52^ Despite extensive through propensity score adjustment for many measured confounders and confounder proxies, including previous use of generic and brand medications, it is possible that residual imbalances in socioeconomic status across treatment groups may still exist. Our Medicare data set lacked laboratory results, which limited our ability to match for baseline glucose control and kidney function. However, a previous study has suggested that PS matching on claims-based data could achieve balance in unmeasured characteristics, such as hemoglobin A_1c_ levels.^53^ In addition, along with the changing guidelines, patients with HF were more likely to receive SGLT-2is in more recent years.^54,55^ Although our PS model included the year of cohort entry and cardiac and kidney comorbidities, we do not have data on ejection fraction or New York Heart Association functional class. Such potential residual imbalances in HF severity, however, would have disfavored SGLT-2is, resulting in conservative estimates. Second, we chose 2 commonly used second-line novel antidiabetic drug classes—DPP-4is and GLP-1RAs—as active comparators. However, the association between these drug classes and the risk of incident AF remains inconclusive.^18,56,57,58,59^ Beyond antimetabolic effects, GLP-1RAs might prevent AF through an anti-inflammatory effect, inhibition of vascular smooth muscle cell proliferation, and reduction of cardiovascular events.^60,61,62^ However, GLP-1RAs may have a direct effect at the sinus node, increase heart rate, and raise the possibility of an increased risk of AF.^63^ Third, our study had a median follow-up time of less than 1 year. The long-term effect of SGLT-2i use on AF remains undetermined. Nevertheless, the large size of our study population allowed us to generate results with high precision despite the relatively short follow-up duration compared with randomized clinical trials. Fourth, the magnitude of the observed absolute risk reduction in AF associated with SGLT-2i therapy was not large. It may be useful for health care professionals to consider the absolute effect when making comparative therapeutic decisions. Fifth, our findings may not be generalizable to younger individuals with T2D.

## Conclusions

In this large population-based cohort including more than 200 000 PS-matched older adults with T2D, initiating treatment with SGLT-2is was associated with reduction in the risk of incident AF compared with DPP-4is (18%) and GLP-1RAs (10%). Our data suggest that the initiation of SGLT-2i in clinical practice may be beneficial in older adults with T2D who are at risk of AF. Besides proven cardiovascular and kidney function benefits, the potential prevention of SGLT-2is in incident AF among older adults with T2D might be considered.

## Notes

Supplement. **eTable 1.** Definitions of Inclusion and Exclusion Criteria **eTable 2.** Outcome Definitions **eFigure 1.** Flowchart of Patients Included in SGLT-2i Versus DPP-4i Cohort **eFigure 2.** Flowchart of Patients Included in SGLT-2i Versus GLP-1RA Cohort **eTable 3.** Baseline Characteristics of SGLT-2i Versus DPP-4i Initiators Before and After 1:1 Propensity Score Matching **eTable 4.** Baseline Characteristics of SGLT-2i Versus GLP-1RA Initiators Before and After 1:1 Propensity Score Matching **eTable 5.** Reasons for Censoring in 1:1 Propensity Score–Matched Cohorts **eTable 6.** Number of Events, Incidence Rate, Hazard Ratios for Sensitivity Analyses in 1:1 Propensity Score–Matched Cohorts
