# Supplementary material for: Association of Sodium-Glucose Cotransporter-2 Inhibitors With Incident Atrial Fibrillation in Older Adults With Type 2 Diabetes
Source: JAMA Netw Open. 2022 Oct 11;5(10):e2235995. doi: 10.1001/jamanetworkopen.2022.35995 (PMC9554705; doi:10.1001/jamanetworkopen.2022.35995)
Supplement: Supplement. — eTable 1. Definitions of Inclusion and Exclusion Criteria eTable 2. Outcome Definitions eFigure 1. Flowchart of Patients Included in SGLT-2i Versus DPP-4i Cohort eFigure 2. Flowchart of Patients Included in SGLT-2i Versus GLP-1RA Cohort eTable 3. Baseline Characteristics of SGLT-2i Versus DPP-4i Initiators Before and After 1:1 Propensity Score Matching eTable 4. Baseline Characteristics of SGLT-2i Versus GLP-1RA Initiators Before and After 1:1 Propensity Score Matching eTable 5. Reasons for Censoring in 1:1 Propensity Score–Matched Cohorts eTable 6. Number of Events, Incidence Rate, Hazard Ratios for Sensitivity Analyses in 1:1 Propensity Score–Matched Cohorts [file jamanetwopen-e2235995-s001.pdf]

Supplementary Online Content

Zhuo M, D'Andrea E, Paik JM, et al. Association of sodium-glucose cotransporter-2 inhibitors with incident atrial fibrillation in older adults with type 2 diabetes. *JAMA Netw Open*. 2022;5(10):e2235995. doi:10.1001/jamanetworkopen.2022.35995

eTable 1. Definitions of Inclusion and Exclusion Criteria

eTable 2. Outcome Definitions

eFigure 1. Flowchart of Patients Included in SGLT-2i Versus DPP-4i Cohort

eFigure 2. Flowchart of Patients Included in SGLT-2i Versus GLP-1RA Cohort

eTable 3. Baseline Characteristics of SGLT-2i Versus DPP-4i Initiators Before and After 1:1 Propensity Score Matching

eTable 4. Baseline Characteristics of SGLT-2i Versus GLP-1RA Initiators Before and After 1:1 Propensity Score Matching

eTable 5. Reasons for Censoring in 1:1 Propensity Score–Matched Cohorts

eTable 6. Number of Events, Incidence Rate, Hazard Ratios for Sensitivity Analyses in 1:1 Propensity Score–Matched Cohorts

This supplementary material has been provided by the authors to give readers additional information about their work.

© 2022 Zhuo M et al. *JAMA Network Open*.

eTable 1. Definitions of inclusion and exclusion criteria

Criteria	Codes
**Type 2 diabetes**	AF diagnosis code in any inpatient position or ⩾2 outpatient visits<br>ICD-9 diagnosis codes: 250.*0, 250.*2<br>ICD-10 diagnosis codes: E11.***
**Type 1 diabetes**	ICD-9 diagnosis codes: 250.*1, 250.*3<br>ICD-10 diagnosis codes: E10.***
**Secondary diabetes**	ICD-9 diagnosis codes: 249.*<br>ICD-10 diagnosis codes: E08.***, E09.***, E13.***, O99.81*
**Malignancy**	ICD-9 diagnosis codes: 140.**-208.** (except 173.**)<br>ICD-10 diagnosis codes: C00.**-C96.** (except C44.**), D03.**, D45
**Chronic kidney disease stage 5 or dialysis**	ICD-9 diagnosis codes: 585.5, 585.6, V45.1*, V56.1-V56.3*, V56.8<br>ICD-9 procedure codes: 39.95, 54.98<br>ICD-10 diagnosis codes: N18.5, N18.6, Z49.31, Z49.32, Z99.2<br>ICD-10 procedure codes: 3E1M39Z, 5A1D00Z, 5A1D60Z<br>CPT-4/HCPCS: 50360,50365, 50380, 90920, 90921, 90924, 90925, 90935,90937, 90940, 90945, 90947, 90957, 90958, 90959, 90960, 90961, 90962, 90965, 90966, 90969, 90970, 90989, 90993, 90999, 99512, 99559, G0257, G0314, G0315, G0316, G0317, G0318, G0319, G0322, G0323, G0326, G0327, S9335, S9339
**Organ transplant**	ICD-9 diagnosis codes: 996.8*, E878.0, V42.*, V58.44<br>ICD-9 procedure codes: 33.5*, 33.6, 37.51, 41.0*, 46.97, 50.5, 50.51, 50.59, 52.8*, 55.6*<br>ICD-10 diagnosis codes: T86.**, Z94.**, Y83.0, Z48.21, Z48.22, Z48.23, Z48.24, Z48.280, Z48.288, Z48.290, Z48.298<br>ICD-10 procedure codes: 02YA0Z0, 02YA0Z1, 02YA0Z2, 0BYC0Z0, 0BYC0Z1, 0BYC0Z2, 0BYD0Z0, 0BYD0Z1, 0BYD0Z2, 0BYF0Z0, 0BYF0Z1, 0BYF0Z2, 0BYG0Z0, 0BYG0Z1, 0BYG0Z2, 0BYH0Z0, 0BYH0Z1, 0BYH0Z2, 0BYJ0Z0, 0BYJ0Z1, 0BYJ0Z2, 0BYK0Z0, 0BYK0Z1, 0BYK0Z2, 0BYL0Z0, 0BYL0Z1, 0BYL0Z2, 0BYM0Z0, 0BYM0Z1, 0BYM0Z2, 0DY80Z0, 0DY80Z1, 0DY80Z2, 0DYE0Z0, 0DYE0Z1, 0DYE0Z2, 0FSG0ZZ, 0FSG4ZZ, 0FY00Z0, 0FY00Z1, 0FY00Z2, 0FYG0Z0, 0FYG0Z1, 0FYG0Z2, 0TS00ZZ, 0TS10ZZ, 0TY00Z0, 0TY00Z1, 0TY00Z2, 0TY10Z0, 0TY10Z1, 0TY10Z2, 30230AZ, 30230G0, 30230G2, 30230G3, 30230G4, 30230X0, 30230X2, 30230X3, 30230X4, 30230Y0, 30230Y2, 30230Y3, 30230Y4, 30233AZ, 30233G0, 30233G2, 30233G3, 30233G4, 30233X0, 30233X2, 30233X3, 30233X4, 30233Y0, 30233Y2, 30233Y3, 30233Y4,

© 2022 Zhuo M et al. *JAMA Network Open*.

	30240AZ, 30240G0, 30240G2, 30240G3, 30240G4, 30240X0, 30240X2, 30240X3, 30240X4, 30240Y0, 30240Y2, 30240Y3, 30240Y4, 30243AZ, 30243G0, 30243G2, 30243G3, 30243G4, 30243X0, 30243X2, 30243X3, 30243X4, 30243Y0, 30243Y2, 30243Y3, 30243Y4, 30250G0, 30250G1, 30250X0, 30250X1, 30250Y0, 30250Y1, 30253G0, 30253G1, 30253X0, 30253X1, 30253Y0, 30253Y1, 30260G0, 30260G1, 30260X0, 30260X1, 30260Y0, 30260Y1, 30263G0, 30263G1, 30263X0, 30263X1, 30263Y0, 30263Y1, 3E03005, 3E030U0, 3E030U1, 3E03305, 3E033U0, 3E033U1, 3E04005, 3E04305, 3E05005, 3E05305, 3E06005, 3E06305, 3E0J3U0, 3E0J3U1, 3E0J7U0, 3E0J7U1, 3E0J8U0, 3E0J8U1<br>CPT-4/HCPCS: 32851-32854, 33935, 33945, 38240, 38241, 44135, 44136, 47135, 47136, 48554, 48556 CPT-4/HCPCS:, 50360, 50365, 50370, 50380
**Prior atrial fibrillation or suspicion of atrial fibrillation**	ICD-9 diagnosis codes: 427.3*<br>ICD-9 procedure codes: 99.61, 99.62, 99.69<br>ICD-10 diagnosis codes: I48.0, I48.1*, I48.2*, I48.3, I48.4, or I48.9*<br>ICD-10 procedure codes: 02580ZZ, 5A2204Z<br>CPT-4/HCPCS: 92960, 92961, 93651<br>NDC medication: digoxin, amiodarone<br>Any anticoagulant (enoxaparin, warfarin, fondaparinux, argatroban, bivalirudin, dabigatran, rivaroxaban, apixaban) AND any of the following medications: acebutolol, atenolol, betaxolol, bisoprolol, carteolol, esmolol, metoprolol, labetalol, penbutolol, nadolol, nebivolol, pindolol, propranolol, sotalol, carvedilol, diltiazem, verapamil)

© 2022 Zhuo M et al. *JAMA Network Open*.

eTable 2. Outcome definitions

Outcome	Codes
**AF hospitalization**	AF diagnosis code in any inpatient position.
**AF diagnosis**	AF diagnosis code in any inpatient position or ⩾2 outpatient visits<br>ICD-9 codes: 427.3*<br>ICD-10 codes: I48.0, I48.1*, I48.2*, I48.3, I48.4, or I48.9*
**AF treated with medication**	AF diagnosis code in any inpatient or outpatient position combined with any AF medication (anticoagulant or antiarrhythmic) within 30 days.
**Hospitalization for AF**	AF discharge diagnosis codes in the primary position.
**Stroke or transient ischemic attack (stroke/TIA)**	Stroke or TIA discharge diagnosis codes in the primary position.<br>ICD-9 codes:<br>433.*1, 434.*1, 435.*, 436.*<br>ICD-10 codes:<br>I60.**, I61.*, I62.*, I63.*, G43.6*9, G45.*, G46.3, G46.4
**Hospitalization for heart failure (HHF)**	Heart failure discharge diagnosis codes in the primary position.<br>ICD-9 codes: 398.91, 402.*1, 404.*1, 404.*3, 428.**<br>ICD-10 codes: I09.81, I11.0, I13.0, I13.2, I50.**
**AF hospitalization censored for HF**	AF diagnosis code in any inpatient position censored for heart failure discharge diagnosis codes in the primary position.
**Hospitalization for AF and heart failure (AF + HF)**	AF and heart failure discharge diagnosis codes in any position.
**Herpes zoster**	Herpes zoster diagnosis code in any inpatient or outpatient position.<br>ICD-9 DX: 053.**<br>ICD-10 DX: B02.**

Abbreviations: AF, atrial fibrillation; HF, heart failure.

© 2022 Zhuo M et al. *JAMA Network Open*.

eFigure 1. Flowchart of patients included in SGLT-2i versus DPP-4i cohort.

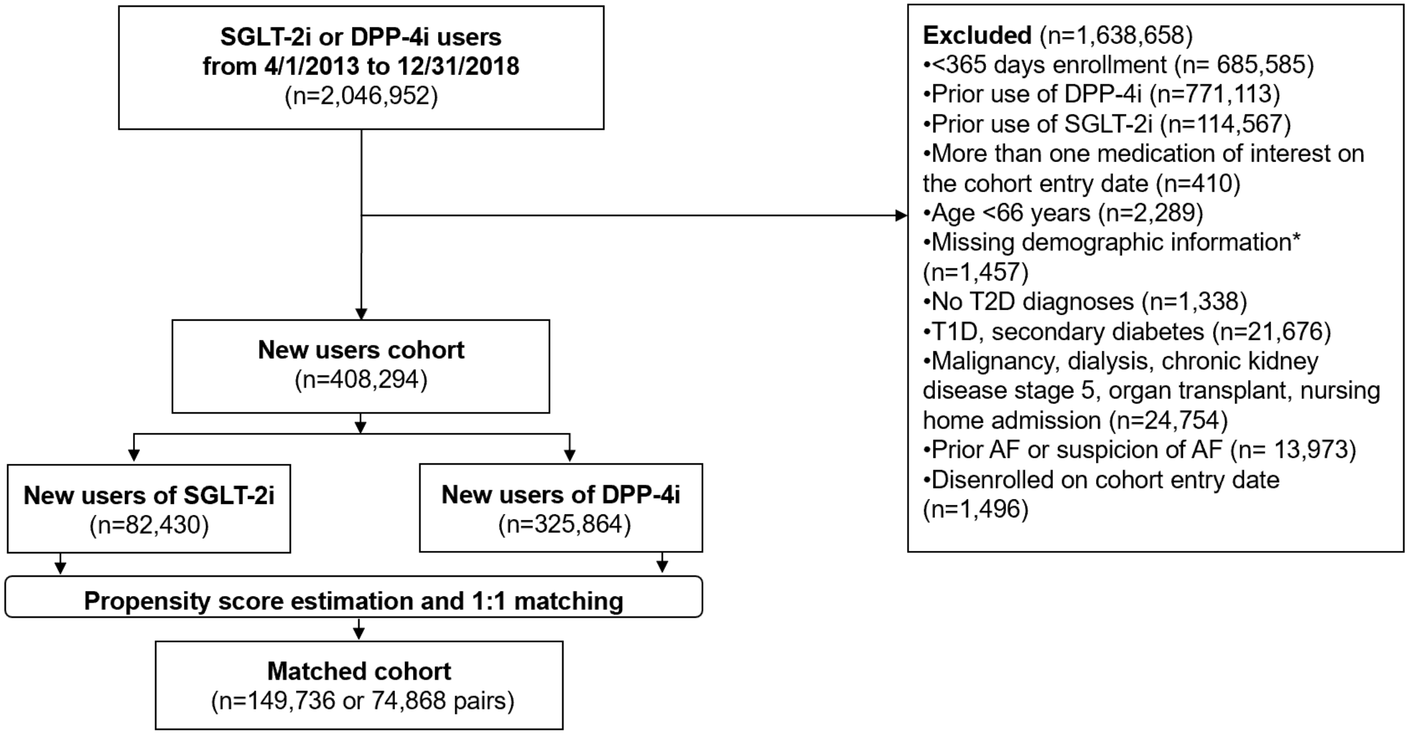

Abbreviations: SGLT-2i, sodium-glucose cotransporter-2 inhibitors; DPP-4i, dipeptidyl peptidase-4 inhibitors; T2D, type 2 diabetes; T1D, type 1 diabetes; AF, atrial fibrillation.

*Demographic information includes age, gender, and race. A total of 1,457 patients with missing race information were excluded.

© 2022 Zhuo M et al. *JAMA Network Open*.

eFigure 2. Flowchart of patients included in SGLT-2i versus GLP-1RA cohort.

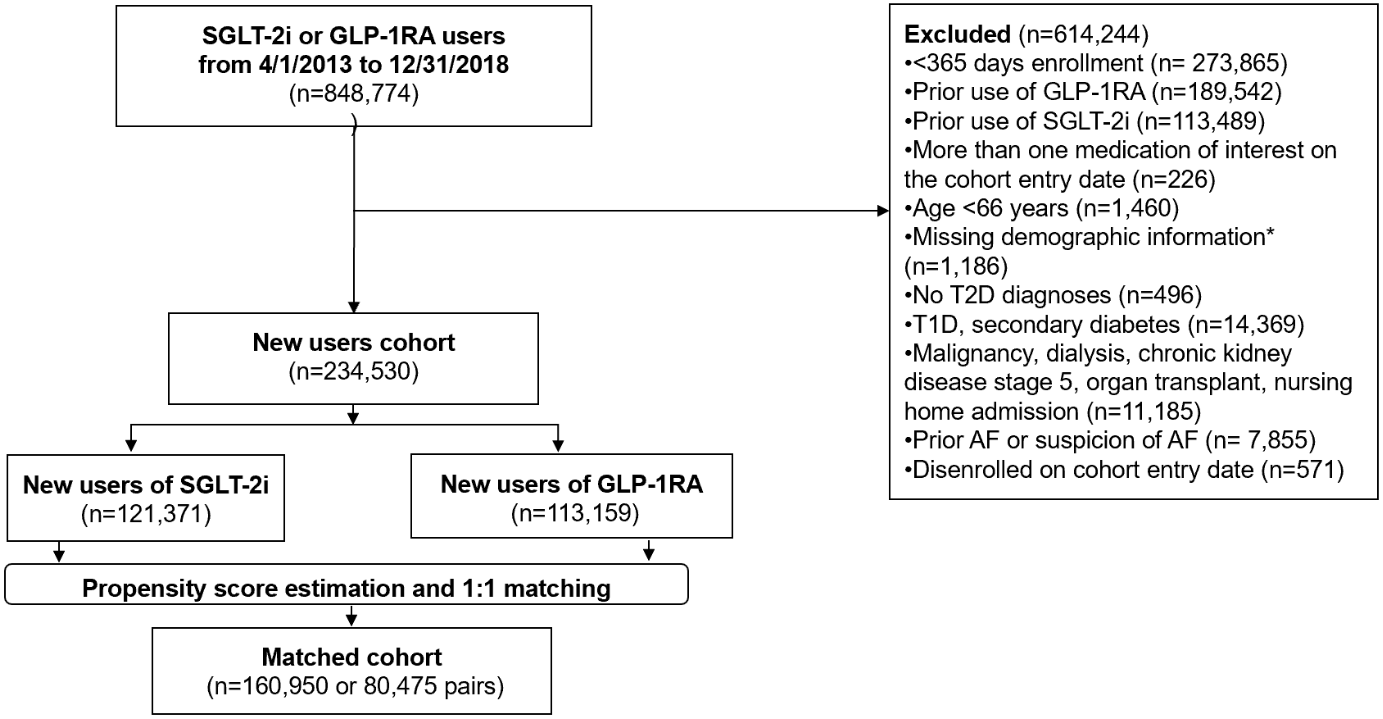

Abbreviations: SGLT-2i, sodium-glucose cotransporter-2 inhibitors; GLP-1RA, glucagon-like peptide-1 receptor agonist; T2D, type 2 diabetes; T1D, type 1 diabetes; AF, atrial fibrillation.

*Demographic information includes age, gender, and race. A total of 1,186 patients with missing race information were excluded.

© 2022 Zhuo M et al. *JAMA Network Open*.

eTable 3. Baseline characteristics of SGLT-2i versus DPP-4i initiators before and after 1:1 propensity score matching.

Characteristics	Before matching			After matching	
	**SGLT-2i (n=82,430) Patients, No. (%)**	**DPP-4i (n=325,864) Patients, No. (%)**	**St. D**	**SGLT-2i (n=74,868) Patients, No. (%)**	**DPP-4i (n=74,868) Patients, No. (%)**
Age, mean (SD), years	71.6 (4.9)	74.2 (6.5)	-0.45	71.85 (5.0)	71.7 (5.1)
Male	40,576 (49.2)	136,293 (41.8)	0.15	36,302 (48.5)	36,398 (48.6)
Race					
Black	6,240 (7.6)	36,951 (11.3)	-0.13	5,904 (7.9)	5,985 (8.0)
White	68,798 (83.5)	242,347 (74.4)	0.22	61,904 (82.7)	61,794 (82.5)
Other[a]	7,392 (9.0)	46,566 (14.3)	-0.17	7,060 (9.4)	7,089 (9.5)
Region					
Northeast	13,960 (16.9)	60,119 (18.4)	-0.04	12,737 (17.0)	12,581 (16.8)
South	36,575 (44.4)	139,050 (42.7)	0.03	33,231 (44.4)	33,223 (44.4)
Midwest	16,825 (20.4)	66,741 (20.5)	0.00	15,297 (20.4)	15,405 (20.6)
West	15,070 (18.3)	59,954 (18.4)	0.00	13,603 (18.2)	13,659 (18.2)
Year of cohort entry					
2013	1,410 (1.7)	45,779 (14.0)	-0.47	1,409 (1.9)	1,249 (1.7)
2014	9,711 (11.8)	67,628 (20.8)	-0.25	9,233 (12.3)	8,922 (11.9)
2015	17,878 (21.7)	65,401 (20.1)	0.04	16,485 (22.0)	16,430 (21.9)
2016	15,572 (18.9)	54,115 (16.6)	0.06	14,329 (19.1)	14,480 (19.3)
2017	18,672 (22.7)	49,235 (15.1)	0.20	16,734 (22.4)	16,903 (22.6)
2018	19,187 (23.3)	43,706 (13.4)	0.26	16,678 (22.3)	16,884 (22.6)
**Diabetes related comorbidities**					
Diabetic nephropathy[b]	9,246 (11.2)	45,357 (13.9)	-0.08	8,293 (11.1)	8,333 (11.1)
Diabetic neuropathy[c]	21,116 (25.6)	73,995 (22.7)	0.07	18,495 (24.7)	18,420 (24.6)
Diabetic retinopathy[d]	11,365 (13.8)	39,415 (12.1)	0.05	9,930 (13.3)	9,816 (13.1)
Diabetes with other ophthalmic manifestation[e]	9,646 (11.7)	44,826 (13.8)	-0.06	8,792 (11.7)	8,698 (11.6)
Diabetes with peripheral circulatory disorders[f]	2,559 (3.1)	16,099 (4.9)	-0.09	2,373 (3.2)	2,369 (3.2)
Diabetic foot[g]	2,086 (2.5)	9,096 (2.8)	-0.02	1,890 (2.5)	1,852 (2.5)
Osteomyelitis [h]	236 (0.3)	1,087 (0.3)	0.00	220 (0.3)	230 (0.3)
Lower extremity amputation[i]	352 (0.4)	1,620 (0.5)	-0.01	323 (0.4)	328 (0.4)
Erectile dysfunction[j]	3,908 (4.7)	10,683 (3.3)	0.07	3,391 (4.5)	3,452 (4.6)

© 2022 Zhuo M et al. *JAMA Network Open*.

Characteristics	Before matching			After matching	
	**SGLT-2i (n=82,430) Patients, No. (%)**	**DPP-4i (n=325,864) Patients, No. (%)**	**St. D**	**SGLT-2i (n=74,868) Patients, No. (%)**	**DPP-4i (n=74,868) Patients, No. (%)**
Hyperglycemia[k]	29,827 (36.2)	82,197 (25.2)	0.24	25,879 (34.6)	26,023 (34.8)
Hypoglycemia[l]	7,110 (8.6)	26,168 (8.0)	0.02	6,251 (8.3)	6,126 (8.2)
Hyperosmolar hyperglycemic nonketotic syndrome[m]	680 (0.8)	2,440 (0.7)	0.01	598 (0.8)	606 (0.8)
Diabetic ketoacidosis[n]	184 (0.2)	1,128 (0.3)	-0.02	168 (0.2)	166 (0.2)
Other diabetic complications[o]	7,226 (8.8)	23,464 (7.2)	0.06	6,335 (8.5)	6,252 (8.4)
**Other comorbid conditions**					
Hyperthyroidism	862 (1.0)	4,310 (1.3)	-0.03	778 (1.0)	803 (1.1)
Hypothyroidism	20,408 (24.8)	81,385 (25.0)	0.00	18,432 (24.6)	18,330 (24.5)
Other disorders of thyroid gland	6,342 (7.7)	22,073 (6.8)	0.03	5,513 (7.4)	5,482 (7.3)
Chronic kidney disease stages 1-2	3,114 (3.8)	14,633 (4.5)	-0.04	2,817 (3.8)	2,854 (3.8)
Chronic kidney disease stages 3-4	6,214 (7.5)	51,428 (15.8)	-0.26	5,896 (7.9)	5,763 (7.7)
Chronic kidney disease stage unspecified	3,392 (4.1)	30,629 (9.4)	-0.21	3,245 (4.3)	3,162 (4.2)
Acute kidney injury	1,882 (2.3)	18,423 (5.7)	-0.17	1,795 (2.4)	1,756 (2.3)
Hypertension	75,654 (91.8)	301,344 (92.5)	-0.03	68,604 (91.6)	68,639 (91.7)
Hyperlipidemia	71,451 (86.7)	279,659 (85.8)	0.03	64,592 (86.3)	64,573 (86.2)
Stable angina	4,864 (5.9)	17,338 (5.3)	0.03	4,350 (5.8)	4,244 (5.7)
Unstable angina	2,091 (2.5)	8,168 (2.5)	0.00	1,888 (2.5)	1,875 (2.5)
Acute myocardial infarction	1,415 (1.7)	6,474 (2.0)	-0.02	1,282 (1.7)	1,298 (1.7)
Old myocardial infarction	3,671 (4.5)	14,243 (4.4)	0.00	3,297 (4.4)	3,295 (4.4)
Coronary atherosclerosis	23,823 (28.9)	92,224 (28.3)	0.01	21,324 (28.5)	21,302 (28.5)
History of coronary procedure	8,182 (9.9)	29,691 (9.1)	0.03	7,260 (9.7)	7,277 (9.7)
Cardiac conduction disorders[p]	3,523 (4.3)	15,882 (4.9)	-0.03	3,157 (4.2)	3,173 (4.2)
Other cardiac dysrhythmia[q]	7,900 (9.6)	37,310 (11.4)	-0.06	7,227 (9.7)	7,229 (9.7)
Cardiomyopathy	1,943 (2.4)	8,824 (2.7)	-0.02	1,764 (2.4)	1,732 (2.3)
Congestive heart failure	6,427 (7.8)	33,744 (10.4)	-0.09	5,907 (7.9)	5,865 (7.8)
Valve disorders	8,179 (9.9)	38,140 (11.7)	-0.06	7,508 (10.0)	7,449 (9.9)
Other cardiovascular disease[r]	9,162 (11.1)	35,307 (10.8)	0.01	8,292 (11.1)	8,237 (11.0)
Transient ischemic attack	1,866 (2.3)	9,677 (3.0)	-0.04	1,710 (2.3)	1,689 (2.3)
Ischemic stroke	8,446 (10.2)	39,583 (12.1)	-0.06	7,730 (10.3)	7,758 (10.4)

© 2022 Zhuo M et al. *JAMA Network Open*.

Characteristics	Before matching			After matching	
	**SGLT-2i (n=82,430) Patients, No. (%)**	**DPP-4i (n=325,864) Patients, No. (%)**	**St. D**	**SGLT-2i (n=74,868) Patients, No. (%)**	**DPP-4i (n=74,868) Patients, No. (%)**
Hemorrhagic stroke	131 (0.2)	818 (0.3)	-0.02	122 (0.2)	126 (0.2)
Peripheral artery disease	9,310 (11.3)	44,833 (13.8)	-0.08	8,505 (11.4)	8,531 (11.4)
Cerebrovascular procedure	182 (0.2)	780 (0.2)	0.00	160 (0.2)	171 (0.2)
Atherosclerotic cerebrovascular disease	1,531 (1.9)	8,917 (2.7)	-0.05	1,417 (1.9)	1,402 (1.9)
Asthma	7,328 (8.9)	29,113 (8.9)	0.00	6,643 (8.9)	6,763 (9.0)
Chronic obstructive pulmonary disease	9,217 (11.2)	43,309 (13.3)	-0.06	8,543 (11.4)	8,620 (11.5)
Obstructive sleep apnea	13,220 (16.0)	34,438 (10.6)	0.16	11,257 (15.0)	11,375 (15.2)
Pneumonia	3,004 (3.6)	16,165 (5.0)	-0.07	2,766 (3.7)	2,710 (3.6)
Liver disease	3,277 (4.0)	13,614 (4.2)	-0.01	2,971 (4.0)	2,984 (4.0)
Nonalcoholic fatty liver disease	4,345 (5.3)	12,963 (4.0)	0.06	3,814 (5.1)	3,782 (5.1)
Osteoarthritis	22,943 (27.8)	89,115 (27.3)	0.01	20,734 (27.7)	20,679 (27.6)
Urinary tract infection	11,823 (14.3)	63,982 (19.6)	-0.14	10,966 (14.6)	10,942 (14.6)
Kidney stone	3,566 (4.3)	13,473 (4.1)	0.01	3,231 (4.3)	3,258 (4.4)
Urinary stone	225 (0.3)	903 (0.3)	0.00	205 (0.3)	197 (0.3)
Hypertensive nephropathy	4,470 (5.4)	35,146 (10.8)	-0.20	4,218 (5.6)	4,264 (5.7)
Proteinuria	4,193 (5.1)	18,312 (5.6)	-0.02	3,682 (4.9)	3,688 (4.9)
Miscellaneous renal disease[s]	7,026 (8.5)	43,468 (13.3)	-0.15	6,551 (8.8)	6,456 (8.6)
Electrolyte disorders	5,028 (6.1)	31,577 (9.7)	-0.13	4,691 (6.3)	4,643 (6.2)
Edema	9,264 (11.2)	41,807 (12.8)	-0.05	8,386 (11.2)	8,361 (11.2)
Falls	3,375 (4.1)	16,268 (5.0)	-0.04	3,080 (4.1)	3,198 (4.3)
Fractures	1,522 (1.8)	6,779 (2.1)	-0.02	1,382 (1.8)	1,358 (1.8)
Anxiety	9,861 (12.0)	39,256 (12.0)	0.00	9,014 (12.0)	9,109 (12.2)
Depression	12,308 (14.9)	49,277 (15.1)	-0.01	11,148 (14.9)	11,141 (14.9)
Psychosis	690 (0.8)	5,768 (1.8)	-0.09	660 (0.9)	652 (0.9)
Dementia	3,056 (3.7)	23,878 (7.3)	-0.16	2,897 (3.9)	2,823 (3.8)
Delirium	467 (0.6)	3,956 (1.2)	-0.06	450 (0.6)	422 (0.6)
Sleep disorders	8,510 (10.3)	30,932 (9.5)	0.03	7,602 (10.2)	7,520 (10.0)
Alcohol abuse or dependence	933 (1.1)	3,849 (1.2)	-0.01	860 (1.1)	854 (1.1)
Drug abuse or dependence	1,290 (1.6)	4,991 (1.5)	0.01	1,160 (1.5)	1,167 (1.6)
Smoking	15,937 (19.3)	58,784 (18.0)	0.03	14,434 (19.3)	14,490 (19.4)

© 2022 Zhuo M et al. *JAMA Network Open*.

Characteristics	Before matching			After matching	
	**SGLT-2i (n=82,430) Patients, No. (%)**	**DPP-4i (n=325,864) Patients, No. (%)**	**St. D**	**SGLT-2i (n=74,868) Patients, No. (%)**	**DPP-4i (n=74,868) Patients, No. (%)**
Overweight[t]	6,785 (8.2)	23,164 (7.1)	0.04	6,133 (8.2)	6,311 (8.4)
Obesity[u]	28,373 (34.4)	75,834 (23.3)	0.25	24,452 (32.7)	24,383 (32.6)
Sepsis/systemic inflammatory response syndrome	803 (1.0)	5,080 (1.6)	-0.05	760 (1.0)	778 (1.0)
Claims-based frailty index					
Non-frail	27,726 (33.6)	91,412 (28.1)	0.12	25,196 (33.7)	25,506 (34.1)
Pre-frail	47,996 (58.2)	190,650 (58.5)	-0.01	43,438 (58.0)	43,094 (57.6)
Frail	6,708 (8.1)	43,802 (13.4)	-0.17	6,234 (8.3)	6,268 (8.4)
Combined comorbidity index (CCI), mean (SD)	1.0 (1.8)	1.4 (2.2)	-0.03	1.0 (1.8)	1.0 (1.8)
**Use of diabetes drugs**					
Metformin	65,240 (79.1)	244,730 (75.1)	0.10	59,258 (79.1)	59,503 (79.5)
Sulfonylureas	37,666 (45.7)	156,966 (48.2)	-0.05	34,628 (46.3)	34,814 (46.5)
Insulins	24,878 (30.2)	51,343 (15.8)	0.35	20,174 (26.9)	20,112 (26.9)
GLP-1RA	15,355 (18.6)	10,248 (3.1)	0.51	8,686 (11.6)	8,114 (10.8)
Glitazones	9,467 (11.5)	29,658 (9.1)	0.08	8,227 (11.0)	8,213 (11.0)
Meglitinides	1,622 (2.0)	6,525 (2.0)	0.00	1,445 (1.9)	1,450 (1.9)
Alpha glucosidase inhibitors	618 (0.7)	2,265 (0.7)	0.00	545 (0.7)	552 (0.7)
Thiazolidinediones	9,467 (11.5)	29,658 (9.1)	0.08	8,227 (11.0)	8,213 (11.0)
Pramlintide	86 (0.1)	35 (0.0)	0.04	32 (0.0)	30 (0.0)
Number of antidiabetic medications, mean (SD)	2.4 (0.9)	2.2 (0.8)	0.01	2.3 (0.9)	2.3 (0.8)
**Other drugs**					
Antiarrhythmics	102 (0.1)	342 (0.1)	0.00	91 (0.1)	96 (0.1)
Anticoagulants	871 (1.1)	3,487 (1.1)	0.00	796 (1.1)	802 (1.1)
Angiotensin converting enzyme inhibitors	37,886 (46.0)	151,846 (46.6)	-0.01	34,519 (46.1)	34,580 (46.2)
Angiotensin II receptor blockers	30,172 (36.6)	114,663 (35.2)	0.03	26,959 (36.0)	26,801 (35.8)
Beta blockers	34,936 (42.4)	143,120 (43.9)	-0.03	31,674 (42.3)	31,645 (42.3)
Calcium channel blockers	26,372 (32.0)	118,969 (36.5)	-0.09	24,164 (32.3)	23,959 (32.0)
Thiazide	13,137 (15.9)	53,566 (16.4)	-0.01	11,922 (15.9)	11,877 (15.9)
Loop diuretics	11,908 (14.4)	55,928 (17.2)	-0.08	10,765 (14.4)	10,697 (14.3)
Mineralocorticoid receptor antagonists	2,994 (3.6)	11,617 (3.6)	0.00	2,662 (3.6)	2,674 (3.6)
Other potassium-sparing diuretics	2,323 (2.8)	10,090 (3.1)	-0.02	2,146 (2.9)	2,145 (2.9)

© 2022 Zhuo M et al. *JAMA Network Open*.

Characteristics	Before matching			After matching	
	SGLT-2i (n=82,430) Patients, No. (%)	DPP-4i (n=325,864) Patients, No. (%)	St. D	SGLT-2i (n=74,868) Patients, No. (%)	DPP-4i (n=74,868) Patients, No. (%)
Other antihypertensive medications	5,933 (7.2)	30,719 (9.4)	-0.08	5,467 (7.3)	5,496 (7.3)
Sacubitril/valsartan	96 (0.1)	207 (0.1)	0.00	81 (0.1)	90 (0.1)
Nitrates and other antianginal agents	6,861 (8.3)	28,859 (8.9)	-0.02	6,153 (8.2)	6,172 (8.2)
Antiplatelet agents	10,752 (13.0)	46,113 (14.2)	-0.04	9,711 (13.0)	9,777 (13.1)
Statins	64,088 (77.7)	245,786 (75.4)	0.05	57,809 (77.2)	57,683 (77.0)
Other lipid-lowering agents	15,143 (18.4)	55,126 (16.9)	0.04	13,397 (17.9)	13,289 (17.7)
COPD/asthma medications	15,654 (19.0)	62,231 (19.1)	0.00	14,163 (18.9)	14,264 (19.1)
NSAIDs	21,897 (26.6)	86,377 (26.5)	0.00	19,909 (26.6)	19,929 (26.6)
Antiosteoporosis agents	3,768 (4.6)	22,358 (6.9)	-0.10	3,551 (4.7)	3,476 (4.6)
Benzodiazepines	11,339 (13.8)	45,922 (14.1)	-0.01	10,311 (13.8)	10,322 (13.8)
Antidepressants	23,959 (29.1)	90,094 (27.6)	0.03	21,512 (28.7)	21,498 (28.7)
Anxiolytics/hypnotics	6,391 (7.8)	26,121 (8.0)	-0.01	5,816 (7.8)	5,838 (7.8)
Corticosteroids	13,692 (16.6)	53,322 (16.4)	0.01	12,478 (16.7)	12,396 (16.6)
Dementia medications	1,946 (2.4)	17,070 (5.2)	-0.15	1,883 (2.5)	1,847 (2.5)
Antiparkinsonian medications	2,945 (3.6)	11,898 (3.7)	-0.01	2,659 (3.6)	2,639 (3.5)
Antipsychotics	1,875 (2.3)	11,075 (3.4)	-0.07	1,741 (2.3)	1,715 (2.3)
Opioids	27,826 (33.8)	110,934 (34.0)	0.00	25,158 (33.6)	25,157 (33.6)
Gabapentinoids	14,528 (17.6)	56,589 (17.4)	0.01	13,069 (17.5)	13,165 (17.6)
Number of total medications, mean (SD)	12.8 (5.7)	12.5 (5.9)	0.05	12.6 (5.7)	12.6 (5.8)
**Health care utilization**					
Electrocardiogram	36,873 (44.7)	153,447 (47.1)	-0.05	33,390 (44.6)	33,344 (44.5)
Cardiac imaging	19,659 (23.8)	82,183 (25.2)	-0.03	17,791 (23.8)	17,702 (23.6)
Cardiovascular stress test	10,461 (12.7)	37,860 (11.6)	0.03	9,360 (12.5)	9,247 (12.4)
Glucose test	27,077 (32.8)	106,659 (32.7)	0.00	23,980 (32.0)	24,042 (32.1)
HbA1c test order	79,870 (96.9)	310,554 (95.3)	0.08	72,440 (96.8)	72,426 (96.7)
Metabolic panel test	79,193 (96.1)	311,509 (95.6)	0.03	71,881 (96.0)	71,923 (96.1)
Pap smear	4,547 (5.5)	16,187 (5.0)	0.02	4,091 (5.5)	4,188 (5.6)
Prostate cancer screening	22,515 (27.3)	72,399 (22.2)	0.12	20,143 (26.9)	20,338 (27.2)
Colorectal cancer screening	8,673 (10.5)	29,326 (9.0)	0.05	7,723 (10.3)	7,708 (10.3)
Influenza vaccine	51,017 (61.9)	191,989 (58.9)	0.06	45,884 (61.3)	45,944 (61.4)

© 2022 Zhuo M et al. *JAMA Network Open*.

Characteristics	Before matching			After matching	
	**SGLT-2i (n=82,430) Patients, No. (%)**	**DPP-4i (n=325,864) Patients, No. (%)**	**St. D**	**SGLT-2i (n=74,868) Patients, No. (%)**	**DPP-4i (n=74,868) Patients, No. (%)**
Pneumococcal vaccine	17,106 (20.8)	55,356 (17.0)	0.10	15,332 (20.5)	15,427 (20.6)
Emergency department visit	20,285 (24.6)	100,665 (30.9)	-0.14	18,739 (25.0)	18,740 (25.0)
Hospitalization	7,637 (9.3)	45,048 (13.8)	-0.14	7,113 (9.5)	7,148 (9.5)
Internal medicine visit	73,207 (88.8)	289,973 (89.0)	-0.01	66,533 (88.9)	66,620 (89.0)
Cardiologist visit	33,001 (40.0)	133,133 (40.9)	-0.02	29,731 (39.7)	29,597 (39.5)
Endocrinologist visit	17,574 (21.3)	42,072 (12.9)	0.22	13,832 (18.5)	13,600 (18.2)
Nephrologist visit	3,096 (3.8)	28,206 (8.7)	-0.20	2,927 (3.9)	2,958 (4.0)
Number of office visits, mean (SD)	10.5 (7.1)	10.4 (7.5)	0.01	10.3 (7.1)	10.3 (7.3)

Values are numbers (percentages) unless stated otherwise
All standardized differences in post-matching cohorts were less than 0.1

Abbreviations: SGLT-2i, sodium-glucose cotransporter-2 inhibitors; DPP-4i, dipeptidyl peptidase-4 inhibitors; St. D, standardized difference; SD, standard deviation; GLP-1RA, glucagon-like peptide-1 receptor agonist; COPD, chronic obstructive pulmonary disease; NSAIDs, nonsteroidal anti-inflammatory drugs; HbA1c, hemoglobin A1c

[a]Other: Asian, North American Native, Hispanic, and other (plus an unknown category).

[b]Diabetic nephropathy: diabetes with renal manifestations, diabetic nephropathy, et al.

[c]Diabetic neuropathy: diabetes with neurological manifestations, polyneuropathy in diabetes, diabetic polyneuropathy, et al.

[d]Diabetic retinopathy: diabetic retinopathy with or without macular edema, pan retinal photocoagulation, history of vitreous hemorrhage or vitrectomy, intravitreal anti-vascular endothelial growth factor injection, et al.

[e]Diabetes with other ophthalmic manifestation: diabetic cataract, diabetic glaucoma, retinal vein occlusion, age-related macular degeneration, et al.

[f]Diabetes with peripheral circulatory disorders: diabetic peripheral angiopathy without gangrene, diabetic peripheral angiopathy with gangrene, type 2 diabetes mellitus with other circulatory complications, et al.

[g]Diabetic foot: type 2 diabetes mellitus with foot ulcer, non-pressure chronic ulcer of calf, non-pressure chronic ulcer of ankle, non-pressure chronic ulcer of heel, et al.

[h]Osteomyelitis: acute hematogenous osteomyelitis, subacute osteomyelitis, chronic osteomyelitis, et al.

[i]Lower extremity amputation: ankle amputation, foot amputation, below knee amputation, above knee amputation, acquired absence of foot, acquired absence of ankle, acquired absence of leg below knee, acquired absence of leg above knee, et al.

[j]Erectile dysfunction: erectile dysfunction due to arterial insufficiency, unspecified male erectile dysfunction, et al.

[k]Hyperglycemia: type 2 diabetes mellitus with hyperglycemia, unspecified hyperglycemia, et al.

[l]Hypoglycemia: hypoglycemia with coma, hypoglycemia without coma, drug-induced hypoglycemia, et al.

[m]Hyperosmolar hyperglycemic nonketotic syndrome: diabetes with hyperosmolarity, diabetes with hyperosmolarity with coma, et al.

[n]Diabetic ketoacidosis: diabetes with ketoacidosis with coma, diabetes with ketoacidosis without coma, et al.

[o]Other diabetic complications: diabetes with unspecified complication

[p]Cardiac conduction disorders: atrioventricular block, left bundle branch block, right bundle branch block, et al.

© 2022 Zhuo M et al. *JAMA Network Open*.

[q]Other cardiac arrhythmia: ventricular tachycardia, premature beats, et al.
[r]Other cardiac vascular disease: rheumatic heart disease, pericarditis, myocarditis, et al.
[s]Miscellaneous renal insufficiency: gouty nephropathy, nephrotic syndrome, vesicoureteral reflex, et al.
[t]Overweight: body mass index 25.0-29.9
[u]Obesity: morbid obesity due to excess calories, gastric restrictive procedure, insertion of gastric bubble, body mass index 30.0-69.9, body mass index 70 and over, et al.

© 2022 Zhuo M et al. *JAMA Network Open*.

eTable 4. Baseline characteristics of SGLT-2i versus GLP-1RA initiators before and after 1:1 propensity score matching.

Characteristics	Before matching			After matching	
	**SGLT-2i (n=121,371) Patients, No. (%)**	**GLP-1 RA (n=113,159) Patients, No. (%)**	**St. D**	**SGLT-2i (n=80,475) Patients, No. (%)**	**GLP-1 RA (n=80,475) Patients, No. (%)**
Age, mean (SD), years	72.2 (5.3)	71.7 (5.0)	0.10	71.8 (5.1)	71.8 (5.1)
Male	58,808 (48.5)	47,650 (42.1)	0.13	35,977 (44.7)	36,025 (44.8)
Race					
Black	9,484 (7.8)	9,792 (8.7)	-0.03	6,581 (8.2)	6,616 (8.2)
White	96,435 (79.5)	94,426 (83.4)	-0.10	66,883 (83.1)	66,583 (82.7)
Other[a]	15,452 (12.7)	8,941 (7.9)	0.16	7,011 (8.7)	7,276 (9.0)
Region					
Northeast	23,044 (19.0)	18,396 (16.3)	0.07	13,693 (17.0)	13,803 (17.2)
South	51,557 (42.5)	48,735 (43.1)	-0.01	35,018 (43.5)	34,958 (43.4)
Midwest	23,298 (19.2)	25,570 (22.6)	-0.08	17,179 (21.3)	17,091 (21.2)
West	23,472 (19.3)	20,458 (18.1)	0.03	14,585 (18.1)	14,623 (18.2)
Year of cohort entry					
2013	2,373 (2.0)	9,961 (8.8)	-0.30	2,372 (2.9)	2,543 (3.2)
2014	15,544 (12.8)	14,788 (13.1)	-0.01	11,221 (13.9)	11,193 (13.9)
2015	27,362 (22.5)	17,086 (15.1)	0.19	14,529 (18.1)	14,727 (18.3)
2016	23,680 (19.5)	18,609 (16.4)	0.08	14,818 (18.4)	14,832 (18.4)
2017	26,709 (22.0)	22,980 (20.3)	0.04	17,479 (21.7)	17,443 (21.7)
2018	25,703 (21.2)	29,735 (26.3)	-0.12	20,056 (24.9)	19,737 (24.5)
**Diabetes related comorbidities**					
Diabetic nephropathy[b]	13,523 (11.1)	21,798 (19.3)	-0.23	10,960 (13.6)	11,044 (13.7)
Diabetic neuropathy[c]	29,917 (24.6)	34,768 (30.7)	-0.14	21,951 (27.3)	21,972 (27.3)
Diabetic retinopathy[d]	16,363 (13.5)	18,387 (16.2)	-0.08	11,586 (14.4)	11,656 (14.5)
Diabetes with other ophthalmic manifestation[e]	14,862 (12.2)	14,493 (12.8)	-0.02	9,720 (12.1)	9,806 (12.2)
Diabetes with peripheral circulatory disorders[f]	4,369 (3.6)	4,112 (3.6)	0.00	2,646 (3.3)	2,743 (3.4)
Diabetic foot[g]	2,852 (2.3)	3,857 (3.4)	-0.07	2,199 (2.7)	2,174 (2.7)
Osteomyelitis [h]	338 (0.3)	432 (0.4)	-0.02	253 (0.3)	247 (0.3)
Lower extremity amputation[i]	466 (0.4)	746 (0.7)	-0.04	382 (0.5)	389 (0.5)
Erectile dysfunction[j]	5,452 (4.5)	4,657 (4.1)	0.02	3,458 (4.3)	3,492 (4.3)

© 2022 Zhuo M et al. *JAMA Network Open*.

Characteristics	Before matching			After matching	
	SGLT-2i (n=121,371) Patients, No. (%)	GLP-1 RA (n=113,159) Patients, No. (%)	St. D	SGLT-2i (n=80,475) Patients, No. (%)	GLP-1 RA (n=80,475) Patients, No. (%)
Hyperglycemia[k]	41,710 (34.4)	44,522 (39.3)	-0.10	30,568 (38.0)	30,289 (37.6)
Hypoglycemia[l]	9,931 (8.2)	11,511 (10.2)	-0.07	7,230 (9.0)	7,212 (9.0)
Hyperosmolar hyperglycemic nonketotic syndrome[m]	996 (0.8)	1,115 (1.0)	-0.02	713 (0.9)	708 (0.9)
Diabetic ketoacidosis[n]	273 (0.2)	326 (0.3)	-0.02	193 (0.2)	184 (0.2)
Other diabetic complications[o]	10,152 (8.4)	11,814 (10.4)	-0.07	7,509 (9.3)	7,528 (9.4)
**Other comorbid conditions**					
Hyperthyroidism	1,368 (1.1)	1,275 (1.1)	0.00	905 (1.1)	916 (1.1)
Hypothyroidism	29,471 (24.3)	30,570 (27.0)	-0.06	20,777 (25.8)	20,852 (25.9)
Other disorders of thyroid gland	9,051 (7.5)	9,971 (8.8)	-0.05	6,657 (8.3)	6,620 (8.2)
Chronic kidney disease stages 1-2	4,672 (3.8)	5,703 (5.0)	-0.06	3,393 (4.2)	3,449 (4.3)
Chronic kidney disease stages 3-4	9,357 (7.7)	20,665 (18.3)	-0.32	8,636 (10.7)	8,780 (10.9)
Chronic kidney disease stage unspecified	5,183 (4.3)	10,496 (9.3)	-0.20	4,558 (5.7)	4,627 (5.7)
Acute kidney injury	2,867 (2.4)	5,353 (4.7)	-0.12	2,428 (3.0)	2,452 (3.0)
Hypertension	111,490 (91.9)	105,577 (93.3)	-0.05	74,383 (92.4)	74,397 (92.4)
Hyperlipidemia	105,538 (87.0)	98,518 (87.1)	0.00	69,568 (86.4)	69,714 (86.6)
Stable angina	7,161 (5.9)	6,784 (6.0)	0.00	4,701 (5.8)	4,711 (5.9)
Unstable angina	2,881 (2.4)	2,855 (2.5)	-0.01	1,982 (2.5)	1,956 (2.4)
Acute myocardial infarction	1,960 (1.6)	1,898 (1.7)	-0.01	1,293 (1.6)	1,292 (1.6)
Old myocardial infarction	5,009 (4.1)	5,261 (4.6)	-0.02	3,501 (4.4)	3,521 (4.4)
Coronary atherosclerosis	34,227 (28.2)	33,412 (29.5)	-0.03	22,927 (28.5)	22,827 (28.4)
History of coronary procedure	11,199 (9.2)	11,336 (10.0)	-0.03	7,661 (9.5)	7,604 (9.4)
Cardiac conduction disorders[p]	4,956 (4.1)	5,398 (4.8)	-0.03	3,494 (4.3)	3,537 (4.4)
Other cardiac dysrhythmia[q]	11,492 (9.5)	11,737 (10.4)	-0.03	7,904 (9.8)	7,887 (9.8)
Cardiomyopathy	2,765 (2.3)	3,024 (2.7)	-0.03	1,943 (2.4)	1,922 (2.4)
Congestive heart failure	9,253 (7.6)	11,834 (10.5)	-0.10	6,883 (8.6)	6,864 (8.5)
Valve disorders	12,346 (10.2)	12,185 (10.8)	-0.02	8,240 (10.2)	8,240 (10.2)
Other cardiovascular disease[r]	12,978 (10.7)	14,167 (12.5)	-0.06	9,262 (11.5)	9,295 (11.6)
Transient ischemic attack	2,859 (2.4)	2,797 (2.5)	-0.01	1,928 (2.4)	1,917 (2.4)
Ischemic stroke	12,924 (10.6)	12,445 (11.0)	-0.01	8,509 (10.6)	8,496 (10.6)
Hemorrhagic stroke	195 (0.2)	204 (0.2)	0.00	137 (0.2)	147 (0.2)

© 2022 Zhuo M et al. *JAMA Network Open*.

Characteristics	Before matching			After matching	
	**SGLT-2i (n=121,371) Patients, No. (%)**	**GLP-1 RA (n=113,159) Patients, No. (%)**	**St. D**	**SGLT-2i (n=80,475) Patients, No. (%)**	**GLP-1 RA (n=80,475) Patients, No. (%)**
Peripheral artery disease	14,689 (12.1)	14,789 (13.1)	-0.03	9,841 (12.2)	9,853 (12.2)
Cerebrovascular procedure	235 (0.2)	242 (0.2)	0.00	162 (0.2)	154 (0.2)
Atherosclerotic cerebrovascular disease	2,360 (1.9)	2,502 (2.2)	-0.02	1,619 (2.0)	1,635 (2.0)
Asthma	10,328 (8.5)	11,666 (10.3)	-0.06	7,623 (9.5)	7,687 (9.6)
Chronic obstructive pulmonary disease	13,447 (11.1)	15,047 (13.3)	-0.07	9,646 (12.0)	9,629 (12.0)
Obstructive sleep apnea	16,015 (13.2)	22,563 (19.9)	-0.18	13,405 (16.7)	13,478 (16.7)
Pneumonia	4,380 (3.6)	5,109 (4.5)	-0.05	3,232 (4.0)	3,235 (4.0)
Liver disease	5,149 (4.2)	4,493 (4.0)	0.01	3,252 (4.0)	3,247 (4.0)
Nonalcoholic fatty liver disease	6,241 (5.1)	6,327 (5.6)	-0.02	4,427 (5.5)	4,436 (5.5)
Osteoarthritis	33,492 (27.6)	35,439 (31.3)	-0.08	23,872 (29.7)	23,914 (29.7)
Urinary tract infection	18,336 (15.1)	20,703 (18.3)	-0.09	13,304 (16.5)	13,247 (16.5)
Kidney stone	5,240 (4.3)	5,529 (4.9)	-0.03	3,721 (4.6)	3,679 (4.6)
Urinary stone	327 (0.3)	338 (0.3)	0.00	234 (0.3)	233 (0.3)
Hypertensive nephropathy	6,720 (5.5)	13,314 (11.8)	-0.23	5,741 (7.1)	5,884 (7.3)
Proteinuria	5,918 (4.9)	8,516 (7.5)	-0.11	4,548 (5.7)	4,572 (5.7)
Miscellaneous renal disease[s]	10,667 (8.8)	14,678 (13.0)	-0.14	8,219 (10.2)	8,249 (10.3)
Electrolyte disorders	7,569 (6.2)	9,399 (8.3)	-0.08	5,469 (6.8)	5,473 (6.8)
Edema	13,186 (10.9)	16,944 (15.0)	-0.12	10,020 (12.5)	10,050 (12.5)
Falls	5,011 (4.1)	5,881 (5.2)	-0.05	3,738 (4.6)	3,760 (4.7)
Fractures	2,189 (1.8)	2,465 (2.2)	-0.03	1,625 (2.0)	1,604 (2.0)
Anxiety	13,929 (11.5)	14,994 (13.3)	-0.05	10,167 (12.6)	10,192 (12.7)
Depression	17,001 (14.0)	21,035 (18.6)	-0.12	13,323 (16.6)	13,354 (16.6)
Psychosis	1,132 (0.9)	1,137 (1.0)	-0.01	760 (0.9)	761 (0.9)
Dementia	5,222 (4.3)	5,215 (4.6)	-0.01	3,540 (4.4)	3,466 (4.3)
Delirium	747 (0.6)	1,004 (0.9)	-0.03	567 (0.7)	552 (0.7)
Sleep disorders	11,730 (9.7)	13,586 (12.0)	-0.07	8,807 (10.9)	8,895 (11.1)
Alcohol abuse or dependence	1,343 (1.1)	1,085 (1.0)	0.01	832 (1.0)	815 (1.0)
Drug abuse or dependence	1,768 (1.5)	2,141 (1.9)	-0.03	1,340 (1.7)	1,354 (1.7)
Smoking	21,949 (18.1)	23,618 (20.9)	-0.07	15,914 (19.8)	15,822 (19.7)
Overweight[t]	10,395 (8.6)	8,511 (7.5)	0.04	6,522 (8.1)	6,483 (8.1)

© 2022 Zhuo M et al. *JAMA Network Open*.

Characteristics	Before matching			After matching	
	SGLT-2i (n=121,371) Patients, No. (%)	GLP-1 RA (n=113,159) Patients, No. (%)	St. D	SGLT-2i (n=80,475) Patients, No. (%)	GLP-1 RA (n=80,475) Patients, No. (%)
Obesity[u]	36,008 (29.7)	47,069 (41.6)	-0.25	29,408 (36.5)	29,319 (36.4)
Sepsis/systemic inflammatory response syndrome	1,184 (1.0)	1,493 (1.3)	-0.03	856 (1.1)	892 (1.1)
Claims-based frailty index					
Non-frail	41,161 (33.9)	28,770 (25.4)	0.19	23,802 (29.6)	23,779 (29.5)
Pre-frail	69,975 (57.7)	70,055 (61.9)	-0.09	48,716 (60.5)	48,669 (60.5)
Frail	10,235 (8.4)	14,334 (12.7)	-0.14	7,957 (9.9)	8,027 (10.0)
Combined comorbidity index (CCI), mean (SD)	1.0 (1.8)	1.5 (2.0)	-0.26	1.2 (1.9)	1.2 (1.9)
**Use of diabetes drugs**					
Metformin	98,097 (80.8)	80,373 (71.0)	0.23	61,810 (76.8)	61,816 (76.8)
Sulfonylureas	61,481 (50.7)	53,117 (46.9)	0.08	39,270 (48.8)	39,280 (48.8)
Insulins	27,179 (22.4)	46,893 (41.4)	-0.42	25,194 (31.3)	25,180 (31.3)
DPP-4i	52,562 (43.3)	37,515 (33.2)	0.21	29,216 (36.3)	29,545 (36.7)
Glitazones	14,568 (12.0)	12,592 (11.1)	0.03	9,296 (11.6)	9,368 (11.6)
Meglitinides	3,295 (2.7)	2,962 (2.6)	0.01	2,092 (2.6)	2,093 (2.6)
Alpha glucosidase inhibitors	1,295 (1.1)	1,091 (1.0)	0.01	815 (1.0)	773 (1.0)
Thiazolidinediones	14,568 (12.0)	12,592 (11.1)	0.03	9,296 (11.6)	9,368 (11.6)
Pramlintide	69 (0.1)	179 (0.2)	-0.03	68 (0.1)	67 (0.1)
Number of antidiabetic medications, mean (SD)	2.6 (1.0)	2.5 (1.0)	0.10	2.5 (1.0)	2.5 (1.0)
**Other drugs**					
Antiarrhythmics	157 (0.1)	138 (0.1)	0.00	97 (0.1)	106 (0.1)
Anticoagulants	1,160 (1.0)	1,384 (1.2)	-0.02	879 (1.1)	875 (1.1)
Angiotensin converting enzyme inhibitors	55,438 (45.7)	52,356 (46.3)	-0.01	37,156 (46.2)	37,080 (46.1)
Angiotensin II receptor blockers	45,540 (37.5)	43,373 (38.3)	-0.02	30,279 (37.6)	30,455 (37.8)
Beta blockers	51,284 (42.3)	51,533 (45.5)	-0.06	35,054 (43.6)	34,988 (43.5)
Calcium channel blockers	39,955 (32.9)	38,906 (34.4)	-0.03	26,754 (33.2)	26,710 (33.2)
Thiazide	18,248 (15.0)	19,891 (17.6)	-0.07	13,328 (16.6)	13,329 (16.6)
Loop diuretics	16,154 (13.3)	23,385 (20.7)	-0.20	12,985 (16.1)	12,998 (16.2)
Mineralocorticoid receptor antagonists	3,848 (3.2)	5,424 (4.8)	-0.08	3,091 (3.8)	3,094 (3.8)
Other potassium-sparing diuretics	3,307 (2.7)	3,598 (3.2)	-0.03	2,457 (3.1)	2,414 (3.0)
Other antihypertensive medications	8,854 (7.3)	10,281 (9.1)	-0.07	6,393 (7.9)	6,354 (7.9)

© 2022 Zhuo M et al. *JAMA Network Open*.

Characteristics	Before matching			After matching	
	SGLT-2i (n=121,371) Patients, No. (%)	GLP-1 RA (n=113,159) Patients, No. (%)	St. D	SGLT-2i (n=80,475) Patients, No. (%)	GLP-1 RA (n=80,475) Patients, No. (%)
Sacubitril/valsartan	161 (0.1)	129 (0.1)	0.00	95 (0.1)	92 (0.1)
Nitrates and other antianginal agents	10,014 (8.3)	10,317 (9.1)	-0.03	6,781 (8.4)	6,749 (8.4)
Antiplatelet agents	16,331 (13.5)	15,475 (13.7)	-0.01	10,632 (13.2)	10,646 (13.2)
Statins	95,323 (78.5)	89,497 (79.1)	-0.01	63,174 (78.5)	63,169 (78.5)
Other lipid-lowering agents	23,517 (19.4)	21,534 (19.0)	0.01	15,164 (18.8)	15,179 (18.9)
COPD/asthma medications	22,821 (18.8)	24,791 (21.9)	-0.08	16,417 (20.4)	16,481 (20.5)
NSAIDs	33,862 (27.9)	31,080 (27.5)	0.01	22,511 (28.0)	22,456 (27.9)
Antiosteoporosis agents	7,232 (6.0)	5,717 (5.1)	0.04	4,139 (5.1)	4,183 (5.2)
Benzodiazepines	16,211 (13.4)	16,528 (14.6)	-0.03	11,447 (14.2)	11,510 (14.3)
Antidepressants	32,712 (27.0)	39,347 (34.8)	-0.17	25,390 (31.6)	25,417 (31.6)
Anxiolytics/hypnotics	9,427 (7.8)	10,016 (8.9)	-0.04	6,678 (8.3)	6,695 (8.3)
Corticosteroids	19,087 (15.7)	20,029 (17.7)	-0.05	13,624 (16.9)	13,604 (16.9)
Dementia medications	3,836 (3.2)	3,437 (3.0)	0.01	2,422 (3.0)	2,377 (3.0)
Antiparkinsonian medications	4,232 (3.5)	5,149 (4.6)	-0.06	3,201 (4.0)	3,230 (4.0)
Antipsychotics	2,976 (2.5)	3,238 (2.9)	-0.02	2,195 (2.7)	2,152 (2.7)
Opioids	39,350 (32.4)	43,128 (38.1)	-0.12	28,539 (35.5)	28,535 (35.5)
Gabapentinoids	20,895 (17.2)	24,659 (21.8)	-0.12	15,700 (19.5)	15,630 (19.4)
Number of total medications, mean (SD)	12.9 (5.9)	14.3 (6.1)	-0.23	13.6 (6.1)	13.6 (5.8)
**Health care utilization**					
Electrocardiogram	54,545 (44.9)	52,433 (46.3)	-0.03	36,342 (45.2)	36,337 (45.2)
Cardiac imaging	28,983 (23.9)	29,131 (25.7)	-0.04	19,578 (24.3)	19,620 (24.4)
Cardiovascular stress test	15,129 (12.5)	15,008 (13.3)	-0.02	10,263 (12.8)	10,246 (12.7)
Glucose test	39,662 (32.7)	40,988 (36.2)	-0.07	27,364 (34.0)	27,409 (34.1)
HbA1c test order	117,541 (96.8)	109,914 (97.1)	-0.02	78,054 (97.0)	78,042 (97.0)
Metabolic panel test	116,848 (96.3)	108,683 (96.0)	0.02	77,204 (95.9)	77,246 (96.0)
Pap smear	6,613 (5.4)	6,651 (5.9)	-0.02	4,698 (5.8)	4,747 (5.9)
Prostate cancer screening	33,182 (27.3)	25,229 (22.3)	0.12	19,486 (24.2)	19,542 (24.3)
Colorectal cancer screening	12,134 (10.0)	12,068 (10.7)	-0.02	8,435 (10.5)	8,426 (10.5)
Influenza vaccine	74,336 (61.2)	71,535 (63.2)	-0.04	50,077 (62.2)	50,203 (62.4)
Pneumococcal vaccine	24,371 (20.1)	23,241 (20.5)	-0.01	16,654 (20.7)	16,714 (20.8)

© 2022 Zhuo M et al. *JAMA Network Open*.

Emergency department visit	29,627 (24.4)	32,325 (28.6)	-0.10	21,157 (26.3)	21,214 (26.4)
Hospitalization	10,895 (9.0)	12,695 (11.2)	-0.07	7,880 (9.8)	7,888 (9.8)
Internal medicine visit	108,345 (89.3)	100,613 (88.9)	0.01	71,508 (88.9)	71,537 (88.9)
Cardiologist visit	47,681 (39.3)	47,956 (42.4)	-0.06	32,550 (40.4)	32,512 (40.4)
Endocrinologist visit	21,581 (17.8)	29,462 (26.0)	-0.20	17,389 (21.6)	17,450 (21.7)
Nephrologist visit	4,631 (3.8)	11,178 (9.9)	-0.24	4,199 (5.2)	4,376 (5.4)
Number of office visits, mean (SD)	10.4 (7.3)	11.8 (7.9)	-0.18	11.0 (7.5)	11.0 (7.4)

Values are numbers (percentages) unless stated otherwise
All standardized differences in post-matching cohorts were less than 0.1

Abbreviations: SGLT-2i, sodium-glucose cotransporter-2 inhibitors; GLP-1RA, glucagon-like peptide-1 receptor agonist; St. D, standardized difference; SD, standard deviation; DPP-4i, dipeptidyl peptidase-4 inhibitors; COPD, chronic obstructive pulmonary disease; NSAIDs, nonsteroidal anti-inflammatory drugs; HbA1c, hemoglobin A1c

[a]Other: Asian, North American Native, Hispanic, and other (plus an unknown category).

[b]Diabetic nephropathy: diabetes with renal manifestations, diabetic nephropathy, et al.

[c]Diabetic neuropathy: diabetes with neurological manifestations, polyneuropathy in diabetes, diabetic polyneuropathy, et al.

[d]Diabetic retinopathy: diabetic retinopathy with or without macular edema, pan retinal photocoagulation, history of vitreous hemorrhage or vitrectomy, intravitreal anti-vascular endothelial growth factor injection, et al.

[e]Diabetes with other ophthalmic manifestation: diabetic cataract, diabetic glaucoma, retinal vein occlusion, age-related macular degeneration, et al.

[f]Diabetes with peripheral circulatory disorders: diabetic peripheral angiopathy without gangrene, diabetic peripheral angiopathy with gangrene, type 2 diabetes mellitus with other circulatory complications, et al.

[g]Diabetic foot: type 2 diabetes mellitus with foot ulcer, non-pressure chronic ulcer of calf, non-pressure chronic ulcer of ankle, non-pressure chronic ulcer of heel, et al.

[h]Osteomyelitis: acute hematogenous osteomyelitis, subacute osteomyelitis, chronic osteomyelitis, et al.

[i]Lower extremity amputation: ankle amputation, foot amputation, below knee amputation, above knee amputation, acquired absence of foot, acquired absence of ankle, acquired absence of leg below knee, acquired absence of leg above knee, et al.

[j]Erectile dysfunction: erectile dysfunction due to arterial insufficiency, unspecified male erectile dysfunction, et al.

[k]Hyperglycemia: type 2 diabetes mellitus with hyperglycemia, unspecified hyperglycemia, et al.

[l]Hypoglycemia: hypoglycemia with coma, hypoglycemia without coma, drug-induced hypoglycemia, et al.

[m]Hyperosmolar hyperglycemic nonketotic syndrome: diabetes with hyperosmolarity, diabetes with hyperosmolarity with coma, et al.

[n]Diabetic ketoacidosis: diabetes with ketoacidosis with coma, diabetes with ketoacidosis without coma, et al.

[o]Other diabetic complications: diabetes with unspecified complication

[p]Cardiac conduction disorders: atrioventricular block, left bundle branch block, right bundle branch block, et al.

[q]Other cardiac arrhythmia: ventricular tachycardia, premature beats, et al.

[r]Other cardiac vascular disease: rheumatic heart disease, pericarditis, myocarditis, et al.

[s]Miscellaneous renal insufficiency: gouty nephropathy, nephrotic syndrome, vesicoureteral reflex, et al.

[t]Overweight: body mass index 25.0-29.9

[u]Obesity: morbid obesity due to excess calories, gastric restrictive procedure, insertion of gastric bubble, body mass index 30.0-69.9, body mass index 70 and over, et al.

© 2022 Zhuo M et al. *JAMA Network Open*.

eTable 5. Reasons for censoring in 1:1 propensity score–matched cohorts

Reasons for censoring	SGLT-2i versus DPP-4i (n=74,868 pairs)		SGLT-2i versus GLP-1RA (n=80,475 pairs)	
	SGLT-2i Patients, No. (%)	DPP-4i Patients, No. (%)	SGLT-2i Patients, No. (%)	GLP-1RA Patients, No. (%)
**Atrial fibrillation hospitalization**	1,082 (1.4)	1,410 (1.9)	1,175 (1.4)	1,235 (1.5)
**Death**	580 (0.8)	935 (1.2)	638 (0.8)	628 (0.8)
**Initiation of comparator medication**	5,460 (7.3)	4,171 (5.6)	5,232 (6.5)	4,357 (5.4)
**Discontinuation of index exposure**	44,188 (59.0)	42,443 (56.7)	47,429 (58.9)	47,970 (59.6)
**End of study date**	19,233 (25.7)	20,930 (28.0)	21,358 (26.5)	21,859 (27.2)
**End of insurance enrollment**	4,325 (5.8)	4,979 (6.7)	4,643 (5.8)	4,426 (5.5)

Abbreviations: SGLT-2i, sodium-glucose cotransporter-2 inhibitors; DPP-4i, dipeptidyl peptidase-4 inhibitors; GLP-1RA, glucagon-like peptide-1 receptor agonist.

© 2022 Zhuo M et al. *JAMA Network Open*.

eTable 6. Number of events, incidence rate, hazard ratios for sensitivity analyses in 1:1 propensity score–matched cohorts

Sensitivity analyses for AF outcome	SGLT-2i	DPP-4i	SGLT-2i vs DPP-4i	SGLT-2i	GLP-1RA	SGLT-2i vs GLP-1RA
	**N events (IR)**	**N events (IR)**	**HR (95% CI)**	**N events (IR)**	**N events (IR)**	**HR (95% CI)**
Grace period and risk period 30 days	818 (16.0)	1,088 (19.7)	0.81 (0.74, 0.89)	871 (17.5)	901 (16.3)	0.93 (0.84, 1.01)
Grace period and risk period 90 days	1,244 (17.1)	1,626 (21.0)	0.82 (0.76, 0.88)	1,350 (17.4)	1,452 (19.2)	0.90 (0.84, 0.97)
Intention-to-treat (ITT)**,** No. (‰)	1,088 (14.5)	1,268 (16.9)	0.86 (0.79, 0.93)	1,160 (14.4)	1,256 (15.6)	0.92 (0.85, 1.00)
Herpes zoster	827 (12.8)	887 (12.9)	0.99 (0.90, 1.09)	893 (12.9)	823 (12.5)	1.04 (0.94, 1.14)

Values are numbers (percentages) unless stated otherwise

Abbreviations: SGLT-2i, sodium-glucose cotransporter-2 inhibitors; DPP-4i, dipeptidyl peptidase-4 inhibitors; GLP-1RA, glucagon-like peptide-1 receptor agonist; AF, atrial fibrillation; N events, number of events; IR, incidence rate in 1000 patient years; HR, hazard ratio; CI, confidence interval.

© 2022 Zhuo M et al. *JAMA Network Open*.
